# A family of accessibility measures derived from spatial interaction principles

**DOI:** 10.1371/journal.pone.0335951

**Published:** 2025-11-14

**Authors:** Anastasia Soukhov, Rafael H. M. Pereira, Christopher D. Higgins, Antonio Páez

**Affiliations:** 1 McMaster University, School of Earth, Environment and Society, Hamilton, Canada; 2 Institute for Applied Economic Research - Ipea, Data Science Division, Brasília, Brazil; 3 University of Toronto Scarborough, Department of Human Geography, Toronto, Canada; Chang’an University, CHINA

## Abstract

Transportation planning has long prioritized the efficiency of movement. However, the concept of accessibility represents a more comprehensive evolution, shifting focus from movement (i.e., trips) to the potential to spatially interact with desired destinations. Despite growing recognition of accessibility-based planning approaches, the concept remains fragmented, with inconsistent definitions and unclear interpretations. To this end, this paper makes a methodological contribution by specifying a family of accessibility measures that are grounded in the shared ‘gravity-based’ theoretical roots of spatial interaction models, particularly their balancing factors. From this foundation, we outline four members of the family: the ‘unconstrained’ measure (i.e., Hansen-type accessibility), the ‘total-constrained’ measure (i.e., a constrained version of the Hansen-type accessibility), the ‘singly-constrained’ measure (i.e., related to the popular two-step floating catchment approach – 2SFCA), and the ‘doubly-constrained’ measure representing realized access (i.e., equal to the doubly-constrained spatial interaction model). These measures can be interpreted as either the number of accessible opportunities or accessible population (i.e., market potential). A toy example illustrates how they produce interpretable unit-based values, offering a clearer and more coherent basis for accessibility analysis.

## 1 Introduction

In the early twentieth century, the emergence of a transportation planning paradigm focused primarily on mobility cemented major investments in automobile and transportation infrastructure, fostering lower-density sprawl, car-dependent development and entrenching automobility in planning practice [1,2]. Within this new practice, access to destinations was treated as a by-product of movement. Despite continued road and highway expansion, this automobility monoculture has proven ineffective at reducing travel costs or environmental burdens, and has not clearly improved people’s ability to reach destinations [3–5].

In response, transportation researchers have increasingly advocated for the adoption of accessibility as a planning criterion, in contrast to traditional mobility-oriented transportation planning approaches which translate into indicators that benchmark movement (e.g., vehicle kilometres traveled, intersection through traffic) which are not necessarily linked to improved accessibility [6–9]. Accessibility, by contrast, is the “potential of opportunities for [spatial] interaction” [10]. While mobility reflects movement, accessibility captures the combined influence of transport and land use, emphasizing destinations and the potential to reach them [11].

Accessibility research has expanded across diverse domains including: employment [12–16], healthcare [5,17–22], green space [23–25], education [26–28], social contact [29–31], and regional economics [32–35], among many other domains of application. Despite its popularity in scholarly works, accessibility still remains difficult to implement in planning due to definitional inconsistencies [8,36,37] and challenges in interpreting and communicating results [36,38,39].

More specifically, the wide range of accessibility definitions, with novel methods being more sophisticated but less intuitive [37], can further hinder practical uptake [36]. Geurs and van Wee [38] classify accessibility measures into four categories: infrastructure-, place-, person-, and utility-based. Among place-based measures (this work’s focus), variants include gravity-based [10], cumulative opportunity [40], the 2 Step Floating Catchment Area (FCA) approach [17], and a variety of modifications to these approaches e.g., Enhanced 2-Step FCA [41], 3-Stage FCA [18], Modified 2-Step FCA [19], inverse 2-Step FCA [42], and n-steps FCA [25]. While these methods are tailored to address specific research contexts, overall this diversity does not demystify existing questions like those raised by van Wee [36]: How should practitioners interpret differences in accessibility scores between modes, and how should results be communicated?

Rather than propose a new measure, this work argues for a return to the spatial interaction foundations of accessibility. Specifically, we show how the family of spatial interaction models [43] can be reformulated in the context of accessibility, namely as a “family of accessibility measures”. This formulation results in constrained versions of gravity-based accessibility (e.g., [10]), but in units of *spatially reachable opportunities*. This approach offers a direct mathematical link to existing accessibility measures while restoring tangible meaning to zonal values. Instead of abstract proportional scores, constrained accessibility expresses the number of opportunities a population may potentially spatially interact with.

This paper makes two contributions. First, we review how spatial interaction modeling and accessibility share similar origins but have diverged in focus and interpretation. Second, we introduce a family of accessibility measures grounded in spatial interaction principles, including total, singly, and doubly-constrained cases and variants of “accessible opportunities” and “accessible population”. These cases and variants align with common measures such as Hansen-type accessibility [10], competitive accessibility measures such as the 2SFCA method [17,44], and market potential models [45,46].

We contend that accessibility research should re-engage with spatial interaction modeling, particularly the use of Wilson’s [43] system constraints. While spatial interaction models embraced such constraints to improve interpretability, most accessibility models have not. This lack of adoption contributes to fuzziness in current analyses, limiting interpretive clarity to simple proportional comparisons (e.g., “higher-than”, “lower-than”) [47]. Without such constraints, accessibility scores lack clear units and comparability across cities or modes. In contrast, constrained measures yield values that can be tied to tangible values without any post-hoc treatment, theoretically making them more interpretable, communicable, and actionable in planning.

The remainder of this paper proceeds as follows. Sects 2 through 4 trace the historical development of spatial interaction and accessibility research, beginning with Newtonian gravitational analogies and Carey (1858) [48] (Sect 2), moving through early researchers like Ravenstein (1885) [49] to Stewart (1948) [50] who theorized and formalized spatial interaction patterns (Sect 3), and examining how the “gravity-based” accessibility approach in Hansen (1959) [10] became the dominant approach in planning practice (Sect 4). Sect 5 presents the entropy-based family of spatial interaction models in Wilson (1971) [43], explaining how the introduction of “constraints” (based on top-down known information as part of entropy-maximization) produces interpretable, unit-consistent flow estimates. Sect 6 explores why accessibility and spatial interaction literature diverged into separate branches despite their shared conceptual foundations. Sect 7 constitutes the paper’s core contribution: we derive a family of accessibility measures corresponding to different constraints applied – unconstrained, singly-constrained, and doubly-constrained cases – demonstrating how each yields zone-level accessibility values expressed in meaningful units (opportunities or population) rather than unit-inconsistent indices. We illustrate each measure with numerical examples that clarify the practical implications of different constraint assumptions. Sect 8 concludes by discussing how these constrained measures can inform planning decisions and improve clarity in accessibility analysis.

The key insight is that accessibility need not be unit-inconsistent. By building from spatial interaction theory’s constraint framework rather than the gravitational analogy alone, we show how to construct accessibility measures that are simultaneously grounded in behavioural principles (the gravitational analogy) as well as being expressed in interpretable units (opportunities or population) that are sensitive to the known to the region through empirically-set constraints.

## 2 Newtonian’s roots of human spatial interaction research

The patterns of people’s movement in space have been a subject of scientific inquiry for at least a century and a half, from as far back as Henry C. Carey’s *Principles of Social Science* [48]. It was in this work where Carey stated that “man [is] the molecule of society [and their interaction is subject to] the direct ratio of the mass and the inverse one of distance” [51, pp. 37–38]. This statement shows how investigations into human spatial interaction have often been explicitly coloured by the features of Newton’s Law of Universal Gravitation, first posited in 1687’s *Principia Mathematica* and expressed as in Eq 1.

$$F_{ij}\propto \frac{M_{i}M_{j}}{D_{ij}^{2}}$$ (1)

This famous equation expresses that the attractive force *F* between two bodies *i* and *j* is directly proportional to the product of their masses and inversely proportional to the square of the distance between them. As mass increases, so does force; as distance increases, force decreases. However, Eq 1 only demonstrates a *proportional* relationship. To quantify the magnitude (not the *proportional* magnitude) of the force, it must be *constrained* with an empirical constant. This constant *G* converts Eq 1 from an expression of proportionality to the following expression of equality:

$$F_{ij}=G\frac{M_{i}M_{j}}{D_{ij}^{2}}$$ (2)

Where *G* is the gravitational constant – an empirically calibrated value that ensures the model reflects observed forces. Newton’s initial estimate of *G* was based on a speculation but received empirical support after Hutton’s and Cavendish’s experiments in the late 1700s [52,53], which estimated it to within 1% accuracy. That is, it took over a century from the publication of Newton’s *Principia Mathematica* to refine the estimate of the proportionality constant.

While the Newtonian gravitational relationship laid the conceptual groundwork for later empirical studies of human spatial interaction, the majority of these early attempts described a proportional relationship or one arbitrarily set to equality. They did not establish an empirical *G* as in the Newtonian tradition, as will be discussed in the next subsection.

## 3 Early research on human spatial interaction: From Ravenstein (1885) to Stewart (1948)

Henry C. Carey’s *Principles of Social Science* [48] inspired empirical spatial interaction research in different contexts. Namely, a number of researchers theoretically and empirically attempted to characterize human spatial interaction as a force *F* directly proportional to the “masses” *M*_*i*_ and *M*_*j*_ of two locations, and inversely proportional to their separation distance – conceptually parallel to Newtonian gravity, but often omitting a proportionality constant.

Beginning with Ravenstein in the late 1880s, his works proposed some “Laws of Migration” based on his empirical analysis of migration flows in various countries [49,54]. These works posited 1) a directly proportional relationship between migration flows and the attractive size of destinations, and 2) an inversely proportional relationship between the size of flows and the separation between origins and destinations. As with Carey, these propositions echo Newton’s gravitational laws.

Over time, other researchers discovered similar relationships. For example, Reilly [55] formulated a law of retail gravitation, expressed in terms of equal attraction to competing retail destinations that could be understood as ‘potential trade territories’. Later, Zipf proposed a $\frac{P_{1}P_{2}}{D}$ hypothesis for the case of information [56], intercity personal movement [57], and goods movement by railways [58]. The $\frac{P_{1}P_{2}}{D}$ hypothesis stated that the magnitude of flows was proportional to the product of the populations of settlements, and inversely proportional to the distance between them.

Of the researchers cited above, only Reilly and Zipf expressed their hypotheses in mathematical terms. Reilly’s hypothesis was presented in the following form:

$$B_{a}=\frac{(P_{a}P_{T}{)}^{N}}{D_{aT}^{n}}$$ (3)

where *B*_*a*_ is the amount of business drawn to *a* from *T*, *P*_*a*_ and *P*_*T*_ are the populations of the two settlements, and *D*_*aT*_ is the distance between them. Quantity *N* was chosen by Reilly in a somewhat *ad hoc* fashion as 1, and he used empirical observations of shoppers to choose a value of *n* = 2.

Zipf, on the other hand, wrote his hypothesis in mathematical form as:

$$C^{2}=\frac{P_{1}P_{2}}{D_{12}}$$ (4)

where *C* is the volume of goods exchanged between 1 and 2, *P*_1_ and *P*_2_ are the populations of the two settlements, and *D*_12_ is the distance between them.

These early formulations (Eqs 3 and 4) clearly reflect the influence of Newtonian gravity on human spatial interaction theory, revealing a shared mathematical structure across migration, trade, and communication models.

However, a common feature of these early investigations is that none of them included a proportionality constant (similar to *G* in Eq 2), a consistent omission of the empirical calibration necessary to convert these proportional relationships into measurable and comparable quantities. It is only in Stewart (1948) [50] that we find the most explicit connection yet to Newton’s Gravitational law and the use of a proportionality constant. While acknowledging predecessors like Reilly and Zipf, the physicist Stewart was likely the first author to formalize human spatial interaction using an explicit proportionality constant *G*, enabling his formulation to be interpreted as a measurable ‘demographic’ force:

$$F=G\frac{\left(\mu_{1}N_{1})(\mu_{2}N_{2}\right)}{d_{12}^{2}}=G\frac{M_{1}M_{2}}{d_{12}^{2}}$$ (5)

Where:

- *F* is the *demographic force*
- *N*_1_ and *N*_2_ are the numbers of people in groups 1 and 2
- $\mu_{1}$ and $\mu_{2}$ are so-called *molecular weights*, the attractive weight of groups 1 and 2
- $M_{1}=\mu_{1}N_{1}$ and $M_{2}=\mu_{2}N_{2}$ are the demographic masses at 1 and 2
- $d_{12}^{2}$ is the distance between 1 and 2
- And finally proportionality constant *G*

What is notable about Eq 5, however, is that the proportionality constant *G* was specified but “left for future determination” [50, p. 34]. We can infer that it is crucial for ensuring *F* is maintained in some units of demographic force.

In addition to demographic force *F*, Stewart defined a measure of the “potential” of group 2 with respect to group 1. The partial sum of the demographic force experienced by group 1, or the potential number of people from location 2 that could visit location 1, as $V_{1}=G\frac{M_{2}}{d_{12}}$. For a system with more than two population bodies, Stewart formulated the population potential at *i* by summing the contributions from each group *j*, after arbitrarily setting *G* = 1:

$$V_{i}=\sum_{j}M_{j}d_{ij}^{-1}$$ (6)

Where *M*_*j*_ is the demographic mass at location *j* and *d*_*ij*_ is the distance between *i* and *j*. A version of this discrete form is what was used in Hansen [10], going on to become a foundation of modern accessibility definitions, as will be discussed.

Although Stewart’s concept of “social physics” eventually fell out of favour, potentially in part due to inconsistent mathematical notation (e.g., *G* is used as both a proportionality constant p. 34 and then later as *demographic energy* on p. 53.) as well as its racist and unscientific assumptions (e.g., view of humans as particles following physical laws and assumptions of the molecular weight of the average American being one, but “presumably... much less than one....for an Australian aborigine” [p. 35]). However, Stewart’s introduction of a proportionality constant *G* in modeling demographic force marks an important conceptual step: recognizing that moving from proportionality to equality requires empirical calibration. In other words, the addition of *G* shifts results from being abstract indicators of potential (i.e., $\frac{{\text{people}}^{2}}{{\text{distance}}^{2}}$) to having units grounded in consistent, albeit still abstract, quantities (i.e., units of some sort of demographic force).

As will be discussed, when Hansen (1959) [10] later adopted Stewart’s formula (Eq 6) for accessibility, he omitted any mention of *G*, effectively setting it to 1 arbitrarily as Stewart had done. This omission has persisted in accessibility research, leaving a conceptual gap in how such measures are interpreted and compared.

## 4 Hansen’s gravity-based accessibility to today

From Stewart [50], we arrive at 1959 and Walter G. Hansen, whose work proved to be exceptionally influential in the accessibility literature [10]. In this seminal paper, Hansen defined accessibility as the potential of opportunities for interaction... a generalization of the population-over-distance relationship or *population potential* concept developed by Stewart [50]” (p. 73). As well as being a student of city and regional planning at the Massachusetts Institute of Technology, Hansen was also an engineer with the Bureau of Roads, and preoccupied with the power of transportation to shape land uses in a very practical sense. Hansen [10] drew directly from Stewart’s population potential formula (see Eq 6), but left aside the broader (and often problematic) framework of “social physics”.

Hansen recast Stewart’s population potential to reflect accessibility, a model of human behaviour useful to capture regularities in mobility patterns. Hansen replaced *M*_*j*_ in Eq 6 with *opportunities* to derive an *opportunity potential*, or more specifically, a *potential of opportunities for interaction* as $S_{i}=\sum_{j}\frac{O_{j}}{d_{ij}^{\beta }}$. A contemporary rewriting of $d^{-\beta }$ to *f*(*d*_*ij*_) accounts for the variety of impedance functions beyond the inverse power used in the applied literature:

$$S_{i}=\sum_{j}O_{j}f(d_{ij})$$ (7)

*S*_*i*_ in Eq 7 is a measure of the accessibility from zone *i*. This is a function of *O*_*j*_ (the mass of opportunities at *j*), *d*_*ij*_ (the cost, e.g., distance or travel time, incurred to reach *j* from *i*), and *f*(*d*_*ij*_) (a function that modulates the friction of cost). Today, Hansen is frequently cited as the father of modern accessibility analysis (e.g., [59]), and Hansen-type accessibility is commonly referred to as the gravity-based accessibility measure.

**Fig 1:**
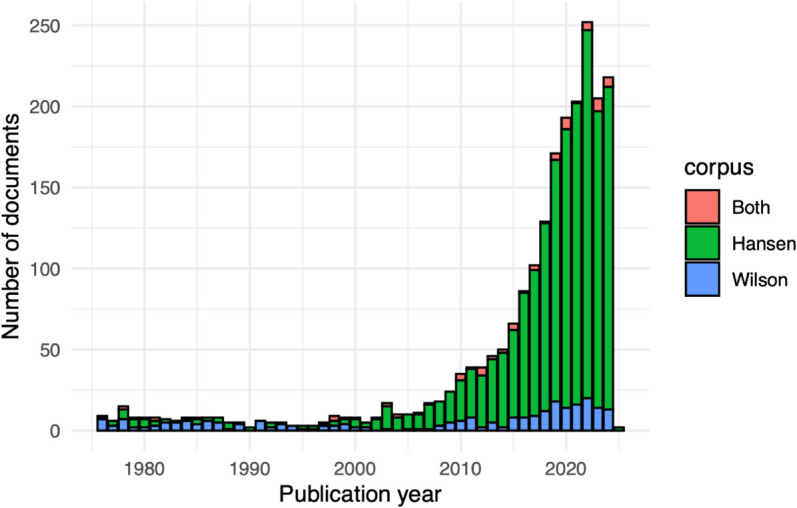
Historical pattern of publication: documents per year.

However, Hansen’s formulation carried forward a crucial omission that continues to affect the literature: the proportionality constant *G* included in Stewart’s original formulation (Eq 6) has vanished entirely. Although Stewart included *G* explicitly (with a note that “*G* [was] left for future determination: a suitable choice of other units can reduce it to unity” [p. 34]). Hansen made no mention of it. As a result, modern accessibility analysis has largely evolved without addressing the constant’s role, leaving *G* effectively and implicitly fixed at 1. This omission has significant implications. Without a proportionality constant, the accessibility formula expresses only a proportional relationship: $S_{i}\propto \sum_{j}g(O_{j})f(d_{ij})$, not one of calibrated equality. Recognition of the nature of this relationship is not common in the literature, but is known, i.e., this proportional equation is shown in Fig 1 in Wu and Levinson [60].

Furthermore, working with a proportional relationship generates fundamental issues in comparability between and, arguably, within studies. Namely, accessibility estimates have no fixed unit, rendering them sensitive to the choice of impedance functions. For instance, if travel cost *d*_*ij*_ is measured in meters, then when the travel impedance function *f*(*d*_*ij*_) equals $d_{ij}^{-\beta }$, the resulting *S*_*i*_ has units of opportunities per ${\text{metres}}^{\beta }$. However, when *f*(*d*_*ij*_) is set to equal $e^{-\beta d_{ij}}$, the units become opportunities per $e^{\beta \text{metres}}$. Such variation impairs comparability across analyses and obscures the meaning of accessibility scores, making them difficult to understand and communicate without post-hoc treatment.

Therefore, in practice, Hansen-type measures are ones of proportionality and are better understood as *ordinal indicators*; they rank accessibility but lack cardinal meaning or consistent units [47]. The continued absence of a sort of proportionality constant *G* leaves a conceptual and practical gap in accessibility analysis: a missing link between theoretical form and empirical measurement that hinders the interpretability of accessibility measures.

## 5 Wilson’s family of spatial interaction models

While accessibility research evolved in North America with Hansen [10], a parallel development was taking place across the Atlantic with Alan G. Wilson. Wilson’s groundbreaking paper [43] advanced a general framework for spatial interaction modeling focused on flows of interaction between places and derived from a non-Newtonian analogy. Additionally, this work was not focused on the ‘potential’ concept as accessibility had been. Wilson [43] formalized the general spatial interaction model through the following equation:

$$T_{ij}=kW_{i}^{\left(1\right)}W_{j}^{\left(2\right)}f(c_{ij})$$ (8)

The model in Eq 8 posits a quantity *T*_*ij*_ that represents a value in a matrix of flows of size *n* × *m*, that is, between i=1,s,n origins and j=1,s,m destinations. The quantities $W_{i}^{\left(1\right)}$ and $W_{j}^{\left(2\right)}$ are proxies for the masses at i=1,s,n origins and j=1,s,m destinations. The super-indices (1) and (2) indicate that these masses can be any number of different things associated with the zones, i.e., $W_{i}^{\left(1\right)}$ could be population at a zone as an origin, and $W_{j}^{\left(2\right)}$ hectares of park space at a zone as a destination. *f*(*c*_*ij*_) is some function of travel cost *c*_*ij*_ which reflects travel impedance.

Of important note for this paper, *k* in Eq 8 acts as a proportionality constant, shifting the equation from a proportional to an equal relationship by incorporating known system totals. In some sense, *k* serves a role similar to the gravitational constant in Newton’s law – it calibrates the model so that outputs match real-world quantities. However, these real-world quantities are not a set empirical constant (like Newton’s *G*) but are instead sensitive to the system and known information about the system.

From the outset, spatial interaction models emphasized interpretability of results [43,61,62]. But unlike earlier approaches that borrowed heuristically from Newtonian gravity (i.e., interaction between masses over distance), Wilson’s innovation was to generalize the model using *entropy maximization*. By maximizing the number of ways individual trip probabilities could be arranged under known constraints, Wilson derived models that estimate *statistical averages* of flows between zones [43,63].

Crucially, to ensure that *T*_*ij*_ in Eq 8 is maintained in units of flow between *i* and *j*, the model moves from proportionality to calibrated equality by incorporating empirical constraints. At a minimum, this requires knowledge of the total number of flows *T* in the system, leading to the basic constraint:

$$\sum_{i}\sum_{j}T_{ij}=T$$ (9)

Additional information can be introduced. For example, when information is available about the total number of flows produced by each origin, $W_{i}^{\left(1\right)}$ in Eq 8, represented as *O*_*i*_, then the following constraint can be used:

$$\sum_{j}T_{ij}=O_{i}$$ (10)

As well, if there is information available about the total number of flows attracted by each destination, $W_{j}^{\left(2\right)}$ is represented as *D*_*j*_ and the following constraint can be used:

$$\sum_{i}T_{ij}=D_{j}$$ (11)

It is also possible to have information about both *O*_*i*_ and *D*_*j*_, in which case both constraints can be imposed on the model at once.

Depending on which of the three system constraints are applied, a family of spatial interaction models can be derived from Eq 8. The proportionality constant *k* is replaced with different *balancing factors*. This change in name is more useful as these factors not only preserve proportionality but also ensure that the predicted flows *T*_*ij*_ align with the known constraints considered in the system. In other words, the different balancing factors adjust the model so that flows become statistical averages consistent with observed origin and/or destination data.

In the framework introduced and inferred from Wilson [43], three types of balancing factors are specified: (1) an unconstrained model that only matches the total volume of interaction *K*, (2) a singly-constrained model (either by origins *A*_*i*_ or destinations *B*_*j*_ – explained below), and (3) a doubly-constrained model that satisfies both.

In the unconstrained model, constraints in Eq 10 and Eq 11 do not hold. In practical terms, this means that the total number of flows predicted by the model must be equal to the sum of all flows from origins *i* to destinations *j*. The balancing factor *K* takes the place of *k* and is equal to the following (as specified in [64] and [65]):

$$K=\frac{T}{\sum_{i}\sum_{j}T_{ij}}$$ (12)

In the singly-constrained model, only constraint Eq 10 or constraint Eq 11 hold. When only Eq 10 holds, entropy maximization leads to the production-constrained singly-constrained version of Eq 8, where the proxy for the mass at the origin $W_{i}^{\left(1\right)}$ is replaced with *O*_*i*_. Also, *k* is replaced with a set of balancing factors specific to origins *A*_*i*_, which ensures that constraint Eq 10 is satisfied (i.e., the sum of predicted flows from one origin going to all destinations must equal the known mass at that origin *O*_*i*_). Satisfying this constraint also implicitly fulfills the total constraint (Eq 9), since the sum of *O*_*i*_ values across all origins equals the total number of flows. *A*_*i*_ takes the following form:

$$A_{i}=\frac{1}{\sum_{j}W_{j}^{\left(2\right)}f(c_{ij})}$$ (13)

The singly-constrained attraction-constrained model is similar to the production-constrained version but from the perspective of the mass at the destination. For the attraction-constrained model, the proxy for the mass at the destination $W_{j}^{\left(2\right)}$ is replaced with *D*_*j*_ in Eq 8, representing the spatial interaction inbound flow. Also, *k* is replaced with a set of destination-specific balancing factors *B*_*j*_ that ensure that Eq 11 is satisfied (hence the total constraint Eq 9 is as well), meaning that the sum of predicted flows going to one destination from all origins must equal the known mass of that destination *D*_*j*_. As before, destination-specific balancing factors *B*_*j*_ were derived by Wilson as:

$$B_{j}=\frac{1}{\sum_{i}W_{i}^{\left(1\right)}f(c_{ij})}$$ (14)

Lastly, the doubly-constrained model is the production-attraction constrained model in Wilson [43]. In this case, both constraints Eq 10 and Eq 11 hold simultaneously. These constraints ensure that the sum of predicted flows from one origin to all destinations, and the predicted flows going to one destination from all origins, must equal the known mass of the origin *O*_*i*_ and of the destination *D*_*j*_. This should hold for all origins and destinations. The resulting model is, in Wilson’s terms, doubly-constrained, and from Eq 8, *k* becomes both *A*_*i*_ and *B*_*j*_ shown in Eq 15, and $W_{i}^{\left(1\right)}$ and $W_{j}^{\left(2\right)}$ is replaced with *O*_*i*_ and *D*_*j*_. Derivation of these models is demonstrated in detail elsewhere (e.g., [62,66]).

$$\begin{array}{c} A_{i}=\frac{1}{\sum_{j}B_{j}D_{j}f(c_{ij})}\\ B_{j}=\frac{1}{\sum_{i}A_{i}O_{i}f(c_{ij})} \end{array}$$ (15)

Wilson’s work is notable for many reasons. Rather than relying on a universal constant like *G* or a scaling factor to balance units, Wilson’s models calibrate interaction flows through known empirical constraints using principles of entropy maximization. This results in interpretable, balanced (given the system knowns are expressed in the constraints), and unit-consistent *ij* flows. The balancing factors themselves have been subject to various interpretations – as terminal costs [67], weighted mean values [61], or as accessibility measures themselves [68] (as suggested in Wilson [43]) and as rents [69] – reflecting ongoing efforts to understand what these mathematical constructs represent behaviourally. In this way, the spatial interaction modeling tradition can be seen to have succeeded where accessibility modeling stalled. While Wilson’s model produces results that are in units of flow tethered to the system of analysis, which facilitates such interpretations, Hansen-type measures (still widely used today in accessibility work) yield outputs that reflect proportional – not equal – relationships and typically lack interpretable units.

Before demonstrating the derivation of the family of accessibility measures that are sensitive to constraints (Sect 7), in the next section, we review how conceptually intertwined the spatial interaction modelling and accessibility literatures are, and where they began to diverge. This investigation sheds light on why accessibility research may have failed to adopt a comparable approach until this paper.

## 6 Accessibility and spatial interaction modelling: Two divergent research streams

Despite their close conceptual ties, the accessibility and spatial interaction modeling literatures have developed along largely separate paths since the 1970s. Hansen’s [10] formulation of accessibility became the method used for decades of work on transport equity, land use analysis, and urban accessibility planning. Meanwhile, Wilson’s [43] entropy-maximizing framework reshaped how spatial interaction models were constructed, particularly in transportation demand forecasting. We argue the framework’s quiet innovation – introducing empirically grounded constraints to shift from proportionality to calibrated equality – made the framework immediately relevant for policy applications as outputs were in tangible units. Yet, this mechanism was never widely adopted in accessibility analysis.

This divergence is especially striking given the context in which both frameworks emerged. As noted in [70], large-scale spatial interaction models (like Wilson’s) responded to important developments at the time, a need “to meet the dictates and needs of public policy for strategic land use and transportation planning”. And these needs were far from trivial: for instance, in the U.S., the Federal-Aid Highway Act of 1956 set in motion the construction of the Interstate Highway System with an eventual budget exceeding $600 billion in today’s dollars [71,72]. In this context, spatial interaction models were incorporated into institutional practices focused on “predict and provide” travel demand forecasting [71,73]. Accessibility analysis, by contrast, remained more conceptually diffuse, focused on indicators of “potential” spatial interaction with opportunities rather than flows that could tangibly guide infrastructure decisions (e.g., roadway capacity expansion, new construction). Whereas spatial interaction modelling became a key element of transportation planning practice, accessibility remained a somewhat more academic pursuit, and the two streams of literature only rarely connected.

To explore why Wilson’s approach may not have crossed over to accessibility modeling sooner, we conducted a review of the literature citing Hansen [10], Wilson [43], or both. This was done using Web of Science’s “Cited References” functionality, and the digital object identifiers of Hansen [10] and Wilson [43]. Only 76 out of the 2,122 documents that emerged from our search of the General Database cite both. The number of articles, by year and if they cite Hansen, Wilson, or both, are shown in Fig 1.

Through the close analysis of why articles that cite both works cite each work, we identify two distinct patterns: one group of articles focused on accessibility, the other on spatial interaction. In examining these groups of articles, we uncover how the relationship between Wilson and Hansen has often been misunderstood, under-explored, or entirely overlooked.

In the first stream of literature – which cite both but are focused more on spatial interaction models – they treat spatial interaction and accessibility as separate but related phenomena. Four subsets of this stream emerge.

First, some of the more early works interpret the spatial interaction model’s balancing factors (Eq 13 or Eq 15) as the inverse of Hansen’s accessibility measure [74–77], likely following Wilson’s own recognition of this similarity between balancing factor *A*_*i*_ and Hansen– type measure *S*_*i*_ on p. 10 in Wilson [43]. In some ways, this relationship has been recognized as a “common sense” approach to incorporating accessibility in the spatial interaction model [78, p. 99], though acknowledgment of its further exploration has been recommended [79].

The second subset of articles within this stream uses both Hansen [10] and Wilson’s [43] framework in conjunction. For instance, some articles argue that spatial interaction models fail to explain certain spatial patterns on their own, for instance, as in Fotheringham [77] who demonstrates how the spatial interaction model may insufficiently explain spatial patterns, and suggests that explicitly defining destinations’ accessibility (Hansen-type accessibility) as a variable within the model may remedy the issue (e.g., the *competition destination* model). Other works take a more applied approach: such as in defining location-allocation problems in operations research [75,80], estimating trips (or some other spatial interaction flows) alongside accessibility (e.g., [81–83]), or considering accessibility as a variable within spatial interaction models, in line with Fotheringham’s [77] demonstration (e.g., [84]).

The third subset of the spatial-interaction focused literature departs from Hansen’s [10] definition, aligning instead with microeconomic or utility-based interpretations of potential spatial interaction e.g., [78,85]. Though across works in this subset, they recognize Hansen-type accessibility as an indicator of ‘potential’ but as a separate but related concept to spatial interaction.

Moving on to the group of accessibility-focused literature that cites both works, we categorize their citation of Wilson [43] within three general groups. Overall, these works do not engage – or only superficially engage – with Wilson [43].

Firstly, there is a group of articles within this stream that cite Wilson [43] exclusively as attribution for using context-dependent travel cost functions. This trend is common: for instance, it is done in the following papers: [11,86–99]. However, these works do not engage with spatial interaction beyond this attribution.

Secondly, a subset of literature explicitly acknowledge the conceptual link between spatial interaction and with Hansen-type accessibility – but only superficially, not going beyond the recognition that they both relate to spatial interaction e.g., [13,60,100–111]. Indeed, while accessibility can be seen as the *potential* for spatial interaction – and Wilson [43] briefly touches on this – such mentions have not resulted in deeper analytical integration of these concepts. Furthermore, some of this literature also occasionally conflates or blurs the distinction entirely, for instance, by co-citing Hansen and Wilson as being ‘gravity models’ (e.g., [96,112–114]). This conflation reveals ongoing murkiness between the distinction of spatial interaction and the *potential for* spatial interaction in the literature.

Thirdly, there is a group of accessibility-focused works that interprets the measure used in Hansen [10] as the singly- or doubly-constrained spatial interaction model’s inverse balancing factor (e.g., [46]). This group often cites the spatial interaction works that make this connection (i.e., the first subset of the first stream of literature) and is especially prominent in the investigation of competitive accessibility topics e.g., [12,115–128]. Only the works of Soukhov et al. [129,130] use Wilson’s [43] balancing factors as a method for maintaining constraints on opportunities within the context of competitive accessibility.

On that note, Soukhov et al., 2023 and 2024 [129,130] introduce the balancing factors as mechanisms to ensure that opportunities at each destination are proportionally allocated to each zone (based on the proportion of population seeking opportunities and the relative travel impedance). This is to ensure that each zonal accessibility value is the sum of this proportional allocation from each destination, and that all zonal values ultimately sum to the total number of opportunities in the region. However, these balancing factors were deduced intuitively. These works did not explicitly state that the mathematical formulations of the equations are effectively equivalent to Wilson’s singly-constrained model (derived from entropy maximization). This equivalence is only discovered in hindsight, as will be demonstrated in the following section. These two works also do not discuss other constrained cases that will also be addressed in this paper.

In sum, despite the interpretative advantages offered by the statistical logic of Wilson [43]’s framework, neither the spatial interaction literature that cites Hansen [10] nor the accessibility literature citing Wilson [43] has meaningfully applied Wilson’s constraint-based intuition to the concept of accessibility. So, in the next section, we do so by demonstrating how the application of Wilson’s framework enables accessibility to move from a proportional relationship to calibrated equality – tying outputs to tangible system knowns. This re-expression of accessibility using constraints yields interpretable, unit-consistent measures of opportunity. This approach takes the same path of entropy-maximization as in Wilson [43], and does not rely on specifying some universal constant *G* like initially suggested in Stewart [50] (recall, Eq 6 which Hansen [10] operationalized).

## 7 A family of accessibility measures: From proportionality to equality

Despite their close conceptual ties, accessibility has not meaningfully absorbed the constraint-based logic of spatial interaction modeling. This section introduces this conceptual connection by defining a family of accessibility measures using Wilson’s approach grounded in statistical mechanics, shifting place-based accessibility ($\overset{`}{a}$ la Hansen [10]) from a relationship describing proportionality to one of equality. This shift addresses the issue of unit interpretability (associated with the proportional nature of Hansen-type accessibility indicators previously outlined).

We propose a revised definition of accessibility considerate of the constraint-based spatial interaction model: *the potential for spatial interaction with opportunities (or population)*. We can specify *k* as a type of proportional allocation factor κ, which incorporates Wilson’s balancing factor(s) to define the *potential for spatial interaction with opportunities* $V_{ij}$ and the *potential for spatial interaction with population M*_*ji*_. In effect, κ is unitless and proportionally allocates (based on the constraints of the case) opportunities (for $V_{ij}$) and population (for *M*_*ji*_). The equations are generally expressed as follows:

$$\begin{array}{c} V_{ij}^{X}=\kappa_{ij}^{X}W_{X}^{\left(2\right)}\\ M_{ji}^{X}={\hat{\kappa }}_{ji}^{X}W_{X}^{\left(1\right)} \end{array}$$ (16)

Where $W_{X}^{\left(2\right)}$ is the mass of the destination (i.e., opportunities *O*_*j*_ or O) and $W_{X}^{\left(1\right)}$ is the mass of the origin (i.e., population *D*_*i*_ or *D*) for either the zone or full region, depending on the case (hence represented by a stand-in *X* subindex). Accessibility flows can also be summarised as a partial sum of the potential at *i* and at *j* to express accessibility at the origin zone or at the destination zone, respectively. This form is common in accessibility research:

$$\begin{array}{c} V_{i}^{X}=\sum_{j}\kappa_{ij}^{X}W_{X}^{\left(2\right)}\\ M_{j}^{X}=\sum_{i}{\hat{\kappa }}_{ji}^{X}W_{X}^{\left(1\right)} \end{array}$$ (17)

**Fig 2:**
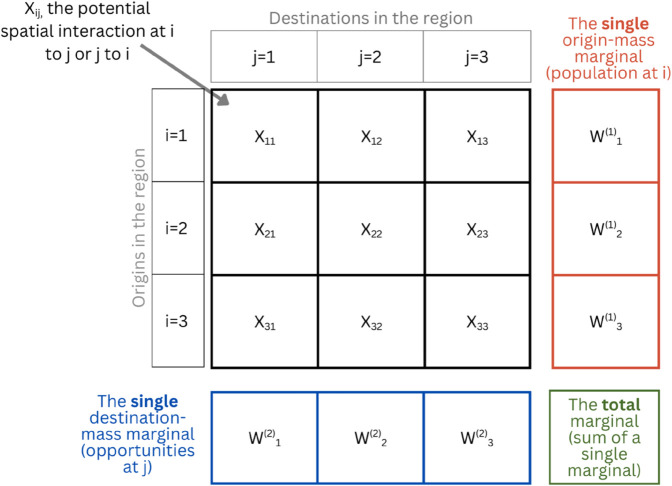
The family of accessibility measures analytical framework.

Fig 2 illustrates our analytical framework using a 3-zone system. The most detailed values, *X*_*ij*_, represent the potential for spatial interaction from origin *i* to destination *j*. Here, *X* stands for all cases and variants to be discussed (e.g., $V_{ij}^{0}$, $M_{ji}^{0}$, $V_{ij}^{T}$, $M_{ji}^{T}$, $V_{ij}^{S}$, $M_{ji}^{S}$, $V_{ij}^{D}$, and $M_{ji}^{D}$). Single marginals show the origin, and destination weights and the total marginal is a sum of these values.

The proportional allocation constant κ takes the form of a balancing factor that varies depending on the constraints applied. Each member of the accessibility measure family is defined by the constraints used, and can be grouped into the following four cases:

**Unconstrained Case ($V_{i}^{0}$, $M_{j}^{0}$)**

- Equivalent to Hansen’s [10] and Reilly’s [55] original formulations, the status quo of accessibility modelling.
- No balancing factors applied; units are in “opportunities-by-impedance” for $V_{i}^{0}$ or “population-by-impedance” for $M_{j}^{0}$.
- No constraints are applied, so values reflect proportionality only and are not calibrated to known system totals.

**Total-constrained Case ($V_{i}^{T}$, $M_{j}^{T}$)**

- Applies a total proportional allocation factor ($\kappa_{ij}^{T}$, ${\hat{\kappa }}_{ji}^{T}$) based only on the total marginal (green box in Fig 2) i.e., total number of opportunities or population in the system. This ensures the sum of all values in the system match the total marginal.
- Units of $V_{i}^{T}$: accessible opportunities from *i*, a value that is total-constrained and linearly proportional to $V_{i}^{0}$.
- Units of $M_{j}^{T}$: accessible population from *j*, a value that is total-constrained and linearly proportional to $M_{j}^{0}$.

**Singly-constrained Case ($V_{i}^{S}$, $M_{j}^{S}$)**

- Applies singly-constrained proportional allocation factors ($\kappa_{ij}^{S}$, ${\hat{\kappa }}_{ji}^{S}$) based on Wilson’s balancing factors (*B*_*j*_, *A*_*i*_) to preserve either the destination-side or origin-side marginal totals (blue and red boxes in Fig 2) i.e., the number of opportunities or population at each zone. Reflects how the literature calculates competitive accessibility.
- Units of $V_{i}^{S}$: accessible opportunities from *i*, a value that is the sum of opportunity supply flows allocated proportionally based on demand at *i*. Mathematically equivalent in per-capita form to 2SFCA [17].
- Units of $M_{j}^{S}$: accessible population from *j*, a value that is the sum of population demand flows allocated proportionally based on supply at *j*.

**Doubly-constrained Case ($V_{ij}^{D}$, $M_{ji}^{D}$)**

- Constrained on both origin and destination sides using both *A*_*i*_ and *B*_*j*_ simultaneously, which can also be expressed as proportional allocation factors ($\kappa_{ij}^{D}$, ${\hat{\kappa }}_{ji}^{D}$); equivalent in interpretation to Wilson’s [43] doubly-constrained spatial interaction model.
- Simultaneous application ensures both the destination-side *and* origin-side marginal totals are maintained (blue and red boxes in Fig 2).
- Interpretable only as *ij* and *ji* flows, since aggregation at *i* and *j* simply reproduces known totals. Represents ‘interaction capacity’ or ‘realized access’ serving as predictions of real interaction flows.

As a summary, each member of the family of accessibility measures is named, explained in plain language, alongside their balancing factor(s), proportional allocation factor(s), and mathematical equation and value interpretations in Table 1.

**Table 1:** Summary of constrained accessibility measure types and interpretations.

Name of Member and Variant	Constraint Explanation and Balancing Factor	Proportional Allocation Factor	Accessibility Measure Equation	Interpretation
Unconstrained Accessible Opportunities ($V_{i}^{0}$) and Unconstrained Accessible Population ($M_{j}^{0}$)	No constraints; marginals not equal to any regional or zonal knowns.	None	$V_{i}^{0}=\sum_{j}D_{j}f(c_{ij})$; $M_{j}^{0}=\sum_{i}O_{i}f(c_{ij})$	Values in various units depending on the impedance and destination-mass (e.g., opportunities × decay) for $V_{i}^{0}$ and impedance and origin-mass (e.g., population × decay) for $M_{j}^{0}$; no total or marginal constraint
Total-constrained Accessible Opportunities ($V_{i}^{T}$) and Total Constrained Accessible Population ($M_{j}^{T}$)	Balancing factors *K*^*T*^ and ${\hat{K}}^{T}$ ensures the sum of *ij* values equals the total marginal, where: $K^{T}=\frac{D}{\sum_{i}V_{i}^{0}}$; ${\hat{K}}^{T}=\frac{O}{\sum_{j}M_{j}^{0}}$	Allocates the total marginal as opportunities *D* based on $\kappa_{ij}^{T}=\frac{W_{j}^{\left(2\right)}f(c_{ij})}{\sum_{i}\sum_{j}W_{j}^{\left(2\right)}f(c_{ij})}$ and as population *O* based on ${\hat{\kappa }}_{ji}^{T}=\frac{W_{i}^{\left(2\right)}f(c_{ij})}{\sum_{i}\sum_{j}W_{i}^{\left(2\right)}f(c_{ij})}$	$V_{i}^{T}=\sum_{j}\kappa_{ij}^{T}D$; $M_{j}^{T}=\sum_{i}{\hat{\kappa }}_{ji}^{T}O$	Values reflect a share of the total opportunities in the region *D* for $V_{i}^{T}$ or total population in the region *O* for $M_{j}^{T}$.
Singly-constrained Accessible Opportunities ($V_{i}^{S}$) and Singly Constrained Accessible Population ($M_{j}^{S}$)	Single balancing factor *B*_*j*_ (for $V_{i}^{S}$) that ensures the destination-mass marginal is constrained, and *A*_*i*_ (for $M_{j}^{S}$) ensures the origin-mass marginal is constrained: $B_{j}=\frac{1}{\sum_{i}W_{i}^{\left(1\right)}f(c_{ij})}$; $A_{i}=\frac{1}{\sum_{j}W_{j}^{\left(2\right)}f(c_{ij})}$	Allocates the single opportunities marginal *D*_*j*_ proportionally based on $\kappa_{ij}^{S}=\frac{W_{i}^{\left(1\right)}f(c_{ij})}{\sum_{i}W_{i}^{\left(1\right)}f(c_{ij})}$ in the case of $V_{i}^{S}$ and the single population marginal *O*_*i*_ proportionally based on ${\hat{\kappa }}_{ji}^{S}=\frac{W_{j}^{\left(2\right)}f(c_{ij})}{\sum_{i}W_{j}^{\left(2\right)}f(c_{ij})}$ in the case of $M_{j}^{S}$.	$V_{i}^{S}=\sum_{j}\kappa_{ij}^{S}D_{j}$; $M_{j}^{S}=\sum_{i}{\hat{\kappa }}_{ji}^{S}O_{i}$	$V_{i}^{S}$ values reflect a share of the opportunities at each destination *D*_*j*_ based on origin population ’demand’ and impedance; $M_{j}^{S}$ values reflect a share of the population at each origin *O*_*i*_ based on destination opportunities ’supply’ and impedance.
Doubly-constrained Access ($V_{ij}^{D}$ or $M_{ij}^{D}$)	Values reflect both single marginals simultaneously, maintained via *A*_*i*_ and *B*_*j*_.	–	$V_{ij}^{D}=A_{i}B_{j}O_{i}D_{j}f(c_{ij})$	The spatial interactions between population and opportunities (i.e., access).

### 7.1 Toy example setup

Consider the simple 3-zone region in Fig 2, where each zone serves as both origin (i) and destination (j). The system includes three inputs: zonal population and opportunities, a zonal cost (travel time) matrix, and three travel impedance functions representing different travel behaviours.

First, Table 2 summarizes population (in 10,000s) and physicians per zone. For context, the Provider-to-Population Ratio (PPR) is 24.5, comparable to Canada’s 2022 PPR of 24.97 physicians per 10,000 [131]. Second, Table 3 shows travel times (minutes); it can be discerned that Zones 1 and 3 are closer to each other than to Zone 2. Zones 1 and 3 together have a population roughly equal to Zone 2 but offer more than twice the physician availability. We interpret Zone 1 as the Urban Edge, Zone 3 as part of the Urban Core, and Zone 2 as Suburban.

**Table 2:** Simple system with three zones (ID 1, 2 and 3). Population is in 10,000 persons and opportunities in number of physicians.

ID (i or j)	Population${\;\;}^{\text{1}}$	Opportunities${\;\;}^{\text{2}}$
1	4	160
2	10	150
3	6	180

**Table 3:** Cost matrix for system with three zones (travel time in minutes).

Origin ID	Destination ID		
	1	2	3
1	10	30	15
2	30	10	25
3	15	25	10

${\;\;}^{\text{1}}$Population is *Wi*^*(1)*^ when used as a proxy for the mass at the origin, and *Oi* when used as a constraint.

${\;\;}^{\text{2}}$Opportunities is *Wj*^*(2)*^ when used as a proxy for the mass at the destination, and *Dj* when used as a constraint.

And third, Eq 18 presents the assumed travel impedance functions reflecting three different travel behaviours. A helpful analogy may be tying travel behaviour to the used mode’s mobility potential, i.e., the most decaying travel behaviour ($f_{1}(c_{ij})$) would assume all travel in the region being done by foot, while calculating accessibility assuming the least decay ($f_{3}(c_{ij})$) would assume unfettered automobility.

$$\begin{array}{c} f_{1}(c_{ij})=\frac{1}{c_{ij}^{3}}\\ f_{2}(c_{ij})=\frac{1}{c_{ij}^{2}}\\ f_{3}(c_{ij})=\frac{1}{c_{ij}^{0.1}} \end{array}$$ (18)

Any set of concepts representing population, opportunities, and their associated travel behaviour, whether representing the entire region uniformly (as will be demonstrated) or representing specific subgroups, can be substituted into our simple toy example. The purpose of the following simple example is to demonstrate the calculation and interpretation of the four accessibility measure variants. As well, the family of measures proposed will be available in the accessibility R package for convenient use in future studies.

### 7.2 Unconstrained accessibility

In $V_{i}^{0}$, no proportional allocation factor is defined; simply *f*(*c*_*ij*_) is used to weight the number of opportunities at each *j* and the weighted values for eachj are summed for each *i*, yielding an expression identical to Hansen’s accessibility *S*_*i*_ [10], the current standard practice in accessibility measurement:

$$V_{i}^{0}=\sum_{j}V_{ij}^{0}=\sum_{j}W_{j}^{\left(2\right)}f(c_{ij})=S_{i}$$ (19)

However, $\sum_{i}V_{i}^{0}$ generally does not equal the total opportunities *O*, so units here are ‘opportunities weighted by travel impedance’ and lack meaningful scaling or direct interpretability. Comparing values across different decay functions or contexts (i.e., different numbers of zones) is therefore limited to ordinal statements (more vs. less), not intervals or ratios (i.e., the magnitude of differences).

For example, Table 4 shows $V_{i}^{0}$ under each decay function. Comparing across decay types is meaningless in absolute terms. For instance, the difference in zone 1 (edge of urban core)’s accessibility under *f*_3_ vs *f*_1_ is 370.92, but in what units? These two values are a product of different impedance functions (${\text{physicians-minute}}^{-0.1}$ and ${\text{physicians-minute}}^{-3}$), making the direct comparison uninterpretable (and arguably incorrect). The fundamental uninterpretability of what is an *opportunity-weighted-travel-impedance* unit remains.

**Table 4:** Simple system: Unconstrained accessibility.

Origin	V${\;\;}_{\text{i}}^{0}$		
	f_1_ (c${\;\;}_{\text{ij}}$) = 1/c${\;\;}_{\text{ij}}^{3}$	f_2_ (c${\;\;}_{\text{ij}}$) = 1/c${\;\;}_{\text{ij}}^{2}$	f_3_ (c${\;\;}_{\text{ij}}$) = 1/c${\;\;}_{\text{ij}}^{0.1}$
	units: *physicians-minute*^*−3*^	units: *physicians-minute*^*−2*^	units: *physicians-minute*^*−0.1*^
1	0.219	2.567	371.143
2	0.167	1.966	363.479
3	0.237	2.751	373.738
Sum	0.6233422	7.283556	1108.361

As the different impedance functions represent different travel behaviours, comparing the raw unconstrained accessibility values across groups is meaningless beyond notions of higher or lower. While one could attempt to adjust the units post-calculation (e.g., scaling, population normalization) or select impedance functions to facilitate comparison across scenarios (potentially at the expense of accurately reflecting travel behaviour), such adjustments may introduce bias. The unconstrained scores are best used for ranking within a single context.

The next sections introduce constraints to calibrate these measures for better interpretability and comparability, applying each to this example in turn.

### 7.3 Total-constrained accessibility

The total-constrained accessibility case can be interpreted in a few ways. In the one that connects to the status quo: the total balancing factor proportionally adjusts unconstrained zonal accessibility values $V_{i}^{0}$ so their total sum of $V_{i}^{0}$ matches a known system total – either total opportunities or total population. Another interpretation is in reformulating the equation to use a proportional allocation constant based on the total balancing factor. The proportional allocation constant distributes opportunities (or population) proportionally by travel impedance.

In both formulations, all zonal values become a proportion of a known system total, be it the regional opportunities or regional population, depending on the variant.

We define two variants for this case:

- $V_{i}^{T}$: accessibility is constrained by the total number of opportunities (total-constrained accessible opportunity), which is interpreted as Hansen’s accessibility with a constraining constant, and
- $M_{j}^{T}$: where *i* and *j* of the first variant are transposed, yielding a measure constrained by the total population and to be interpreted as constrained ‘market potential’.

#### 7.3.1 Total-constrained accessible opportunities: Hansen’s accessibility with a total constraint.

In the total-constrained case, accessibility is expressed as a share of the total number of opportunities in region *D*, allocated based on travel impedance. The total-constrained accessibility from *i* to *j* takes the form:

$$V_{ij}^{T}=\kappa_{ij}^{T}D$$ (20)

This formulation satisfies the total constraint, analogous to the one in the Wilson framework:

$$\sum_{i}\sum_{j}V_{ij}^{T}=D$$ (21)

Next, the proportional allocation factor $\kappa_{ij}^{T}$ determines the share of total opportunities assigned to each origin–destination pair, based on the relative proportion of opportunities-weighted travel impedance:


$$\kappa_{ij}^{T}=\frac{W_{j}^{\left(2\right)}f(c_{ij})}{\sum_{i}\sum_{j}W_{j}^{\left(2\right)}f(c_{ij})}$$

This renders $V_{i}^{T}$ (equal to $\sum_{j}V_{ij}^{T}$) into units of opportunities (e.g., physicians), and allows direct interpretation and comparison of results between zones and scenarios.

Alternatively, this formulation can be rewritten to be expressed using a total-constrained balancing factor *K*^*T*^, which scales Hansen’s unconstrained accessibility $V_{i}^{0}$ to meet the total opportunity constraint:

$$V_{i}^{T}=\sum_{j}V_{ij}^{T}=K^{T}\sum_{j}W_{j}^{\left(2\right)}f(c_{ij})=K^{T}V_{i}^{0}$$ (22)

Where the total-constrained balancing factor *K*^*T*^ is:

$$K^{T}=\frac{D}{\sum_{i}\sum_{j}W_{j}^{\left(2\right)}f(c_{ij})}$$ (23)

This expression is consistent with Wilson’s entropy-maximizing framework and analogous to the total flow spatial interaction model (e.g., Equation 2.11 in [64]).

In summary, $\kappa_{ij}^{T}$ proportionally allocates the total number of opportunities *D* to each origin–destination pair based on relative opportunity-weighted travel impedance. These values can be aggregated across destinations to obtain total-constrained accessibility at each origin. Alternatively, the measure can be expressed using the balancing factor *K*^*T*^, demonstrating that it is algebraically proportional to unconstrained accessibility $V_{i}^{0}$, but with interpretable units (i.e., opportunities). This allows for meaningful comparisons of differences across zones and travel behaviour scenarios.

Referring back to our simple numeric example, *K*^*T*^ for the highest decay travel scenario $f_{1}(c_{ij})=1/c_{ij}^{3}$ would then be:


$$K^{T}=\frac{D}{\sum_{i}\sum_{j}W_{j}^{\left(2\right)}f(c_{ij})}=\frac{490}{0.6233}=786.085$$

*K*^*T*^ for other decay scenarios are calculated similarly in code. Applying each *K*^*T*^ to the unconstrained values $V_{i}^{0}$ yields total-constrained accessibility values (Table 5), all in units of physicians.

**Table 5:** Simple system: Total-constrained accessible opportunities.

Origin	V${\;\;}_{\text{i}}^{T}$		
	f_1_ (c${\;\;}_{\text{ij}}$) = 1/c${\;\;}_{\text{ij}}^{3}$	f_2_ (c${\;\;}_{\text{ij}}$) = 1/c${\;\;}_{\text{ij}}^{2}$	f_3_ (c${\;\;}_{\text{ij}}$) = 1/c${\;\;}_{\text{ij}}^{0.1}$
	units: *physicians*	units: *physicians*	units: *physicians*
1	172.065	172.672	164.080
2	131.627	132.247	160.692
3	186.308	185.081	165.228
Sum	490	490	490

Compared to the unconstrained case, values now sum to the known regional total *D*, allowing interpretation of absolute and relative differences across zones and travel scenarios. For example, in the highest decay case, Zone 1 (Urban Edge) captures an intermediate number of physicians (172.065), like in the unconstrained accessibility case. However, unlike in the unconstrained case, we can say that this value is out of the 490 physicians in the region, which allows us to deduce that zone 1 captures 1.307 and 0.924 times more than zone 2 and 3. Values for the lesser decay ($f_{2}(c_{ij}))$ and lowest decay $\left(f_{3}(c_{ij}\right)$) scenarios are calculated separately, with decay scenario values also summing to 490 physicians accessible in the region.

One can also directly compare values at a specific zone, across travel impedance scenarios, due to the consistent units. As the decay scenario decreases, all zones become more accessible to each other and the differences between pairs diminish (i.e., in $f_{3}(c_{ij})$ each zone captures close to an average amount of physicians, a third of 490 or ~163). In terms of proportional magnitude, this can also be observed in the unconstrained measure for this scenario. However, for the total-constrained measure, this plateauing of results has meaning. In fact, each zone is allocated an average of the total amount in the region, as a result of the total-constrained proportional allocation factor.

However, what’s notable is how zones change between scenarios. For instance, Zone 1’s share only declines slightly – these declines are outpaced by Zone 2’s relative gains. This shift reflects how $\kappa_{ij}^{T}$ redistributes opportunities in proportion to impedance under different travel behaviours.

The total-constrained accessibility measure resolves the interpretability issue of Hansen’s accessibility (i.e., unconstrained accessibility) by grounding values in a meaningful total, enabling robust comparisons across zones and scenarios, but also by keeping values proportional to $V_{i}^{0}$, so interpretation is similar.

#### 7.3.2 Total-constrained accessible population: Reilly’s potential trade territories with a total constraint.

This second total-constrained variant is a transpose of the opportunity-constrained formulation, switching indices *i* and *j* to yield a measure of market potential – the number of people who can spatially interact with a destination.

Though not outlined in the “Unconstrained accessibility” section, the unconstrained form aligns with Reilly’s “potential trade territories” [55] and Harris’ and Vickerman’s formulations of regional market potential [45,46]. In its unconstrained form, market potential has also been more recently used to estimate potentially accessible populations following infrastructure investments (e.g., [132–134]). Market potential can also be thought of as a form of *passive accessibility*, indicating the number of people that can reach each destination.

However, like $V_{ij}^{0}$, issues of unit interpretability arise in unconstrained market potential $M_{j}^{0}$. To address this, we introduce the total-constrained accessible population measure $M_{ji}^{T}$, which allocates the total population *O* across all origin-destination pairs proportionally:

$$M_{ji}^{T}=\kappa_{ji}^{T}O$$ (24)

Subject to the total constraint::

$$\sum_{i}\sum_{j}M_{ji}^{T}=O$$ (25)

Next, ${\hat{\kappa }}_{ij}$ is the total-constrained proportional allocation factor, a dimensionless term which distributes population based on impedance-weighted accessibility:


$${\hat{\kappa }}_{ij}^{T}=\frac{W_{i}^{\left(1\right)}f(c_{ij})}{\sum_{i}\sum_{j}W_{i}^{\left(1\right)}f(c_{ij})}$$

This renders $M_{j}^{T}$ (equal to $\sum_{j}M_{ji}^{T}$) into units of population. Alternatively, this formulation can be rewritten to be expressed using a total-constrained balancing factor ${\hat{K}}^{T}$, which scales unconstrained market potential $M_{j}^{0}$ to meet the total population constraint:

$$M_{j}^{T}=\sum_{i}M_{ji}^{T}={\hat{K}}^{T}\sum_{i}W_{i}^{\left(1\right)}f(c_{ij})={\hat{K}}^{T}M_{j}^{0}$$ (26)

Where the total-constrained balancing factor ${\hat{K}}^{T}$ is:

$${\hat{K}}^{T}=\frac{D}{\sum_{i}\sum_{j}W_{i}^{\left(1\right)}f(c_{ij})}$$ (27)

In summary, ${\hat{\kappa }}_{ij}^{T}$ allocates the total number of population *O* proportionally to each origin–destination pair based on relative population-weighted travel impedance. As well, the measure can be expressed using the balancing factor ${\hat{K}}^{T}$, demonstrating that it is algebraically proportional to unconstrained market potential, but also yielding interpretable units that allow for meaningful comparison.

Returning to the numerical example, the balancing factor ${\hat{K}}^{T}$ is solved for each travel behaviour scenario, and the market potential of each zone $M_{j}^{T}$ is expressed as units of population (e.g., the number of people accessible from each origin at that destination) in Table 6.

**Table 6:** Simple system: Total-constrained accessible population.

Destination	M${\;\;}_{\text{i}}^{\text{S}}$		
	f_1_ (c${\;\;}_{\text{ij}}$) = 1/c${\;\;}_{\text{ij}}^{3}$	f_2_ (c${\;\;}_{\text{ij}}$) = 1/c${\;\;}_{\text{ij}}^{2}$	f_3_ (c${\;\;}_{\text{ij}}$) = 1/c${\;\;}_{\text{ij}}^{0.1}$
	units: *population in 10,000s*	units: *population in 10,000s*	units: *population in 10,000s*
1	5.018	5.447	6.598
2	8.596	7.986	6.717
3	6.386	6.567	6.684
Sum	20	20	20

Readers may note the difference in trends in accessible population (Table 6) and accessible physicians (i.e., the preceding subsection, Table 5).

In Table 5, zones 1-3 represent destinations, and the accessibility values reflect the number of accessible people from the vantage of physicians. Zone 1, in its role as a destination, is no longer intermediately ranked relative to other zones; it now attracts the fewest number of people across all three travel behaviour scenarios. However, similar to the total-constrained opportunity case, as travel decay reduces, the availability of population begins to converge (though Zone 1 continues as the lowest-ranked) for similar reasons. As decay reduces, the population’s travel impedance to all zones becomes more similar, making the relative location of the zones less important and all people in the region more equally accessible.

Like in the total-constrained accessible opportunities variant, the total-constrained accessible population enables direct comparison of raw values, supporting both ordinal and interval interpretations across space and travel behaviour scenarios.

### 7.4 Singly-constrained accessibility

The singly-constrained accessibility case can also be expressed in two variants, each defined by the direction in which a constraint is applied:

- $V_{i}^{S}$: accessibility constrained by opportunities at destinations (singly-constrained accessible opportunities), and
- $M_{j}^{S}$: its transpose, constrained by population at origins (singly-constrained accessible population, or market potential).

Similar to the total-constrained case, the singly-constrained measures adjust unconstrained zonal accessibility values ($V_{i}^{0}\text{or}M_{j}^{0}$) using a balancing factor to satisfy the known system constraint. However, unlike the total constraint (which enforces a regional sum), the singly-constrained case applies a localized constraint at one end of the interaction – either origin or destination.

In the opportunities-constrained variant $V_{i}^{S}$, the balancing factor ensures that only a proportion of opportunities at each destination are allocated to origins, based on their relative demand (population) and travel impedance. This variant mirrors the concept of spatial availability as discussed in Soukhov et al. [129]. In the population-constrained variant $M_{j}^{S}$, the logic is reversed: population at each origin is allocated proportionally across destinations, informed by the distribution of opportunities and impedance.

In both cases, the singly-constrained formulation introduces zonal-level competition, unlike the total-constrained case, which distributes a fixed regional sum. Each zonal accessibility value becomes not only a fraction of the regional total (opportunities or population), but also a balanced sum of interactions, weighted by impedance and relative competition. The result remains in interpretable units – accessible opportunities or accessible population – but reflects these more complex spatial dynamics.

#### 7.4.1 Singly-constrained accessible opportunities: Spatial availability.

In this singly-constrained variant, accessibility is constrained at the destination side: the sum of accessible opportunities allocated from each destination must equal the known number of opportunities *D*_*j*_. This is comparable to the single attraction-constraint (Eq 11) from Wilson’s framework:

$$\sum_{i}V_{ij}^{S}=D_{j}$$ (28)

The underlying spatial interaction model is now the attraction-constrained model, and our accessibility measure becomes:

$$V_{i}^{S}=\sum_{j}B_{j}D_{j}W_{i}^{\left(1\right)}f(c_{ij})$$ (29)

where $W_{i}^{\left(1\right)}$ is a measure of the mass at origin *i* (i.e., the opportunity-seeking population). The corresponding balancing factor, as per Wilson, is:

$$B_{j}=\frac{1}{\sum_{i}W_{i}^{\left(1\right)}f(c_{ij})}$$ (30)

Introducing the balancing factor in Eq 29, we obtain:

$$V_{i}^{S}=\sum_{j}D_{j}\frac{W_{i}^{\left(1\right)}f(c_{ij})}{\sum_{i}W_{i}^{\left(1\right)}f(c_{ij})}$$ (31)

Further, we can express the formula even more simply by defining the following proportional allocation factor:

$$\kappa_{ij}^{S}=\frac{W_{i}^{\left(1\right)}f(c_{ij})}{\sum_{i}W_{i}^{\left(1\right)}f(c_{ij})}$$ (32)

After this, it is possible to rewrite Eq 31 as an origin summary expression of proportionally allocated known opportunities (i.e., *D*_*j*_).

$$V_{i}^{S}=\sum_{j}\kappa_{ij}^{S}D_{j}$$ (33)

This formulation has been referred to as **spatial availability** by Soukhov et al. [129], since it incorporates spatial competition by allocating opportunities based on demand (population), impedance, and the known opportunity totals *D*_*j*_. The dimensionless factor $\kappa_{ij}^{S}$ ensures that each destination’s opportunities are distributed proportionally to origins. As in the total-constrained case, $V_{i}^{S}$ is expressed in the units of accessible opportunities.

Soukhov et al. [129] also showed that the following expression (accessibility per capita) is a constrained version of the popular 2SFCA approach of Shen [44] and Luo and Wang [17]:

$$v_{i}^{S}=\frac{V_{i}^{S}}{W_{i}^{\left(1\right)}}$$ (34)

Returning to the simple numeric example, as an example of the solved *B*_*j*_ for the highest decay travel behaviour $f_{1}(c_{ij})$:


$$\begin{array}{c} B_{j}=\frac{1}{\sum_{i}W_{i}^{\left(1\right)}f(c_{ij})}\\ B_{1}=\frac{1}{\frac{4}{10^{3}}+\frac{10}{30^{3}}+\frac{6}{15^{3}}}=162.6506\\ B_{2}=\frac{1}{\frac{4}{30^{3}}+\frac{10}{10^{3}}+\frac{6}{25^{3}}}=94.9474\\ B_{3}=\frac{1}{\frac{4}{10^{3}}+\frac{10}{25^{3}}+\frac{6}{10^{3}}}=93.9850 \end{array}$$

The balancing factors *B*_*j*_ for the $f_{2}(c_{ij})$ decay group for zones 1, 2 and 3 are 12.857, 8.769 and 10.664, respectively. For the $f_{3}(c_{ij})$ decay group, they are 0.067, 0.066 and 0.066. Using these balancing constants, we can calculate the singly-constrained opportunity accessibility (Table 7).

**Table 7:** Simple system: Singly-constrained accessible opportunities.

Origin	Population (10k)	V${\;\;}_{\text{i}}^{\text{S}}$		
		f_1_ (c${\;\;}_{\text{ij}}$) = 1/c${\;\;}_{\text{ij}}^{3}$	f_2_ (c${\;\;}_{\text{ij}}$) = 1/c${\;\;}_{\text{ij}}^{2}$	f_3_ (c${\;\;}_{\text{ij}}$) = 1/c${\;\;}_{\text{ij}}^{0.1}$
		units: *physicians*	units: *physicians*	units: *physicians*
1	4	133.469	122.255	98.848
2	10	166.781	185.096	241.877
3	6	189.750	182.650	149.275
Sum	—	490	490	490

Imposing the single proportional allocation factor $\kappa_{ij}^{S}$ allows for the comparison of differences and ratios of the accessibility values, like previously discussed in the total-constrained accessible opportunities case. The proportional allocation factor ensures that resulting values are in units of *physicians*, with the impedance units already accounted for in the allocation process.

However, unlike $\kappa_{ij}^{T}$, $\kappa_{ij}^{S}$ introduces zonal competition based on the mass of the origin (population). In the total-constrained case, opportunities are distributed based on impedance alone, regardless of population at *i*. In contrast, the singly-constrained case allocates each zone’s opportunities proportionally across the region based on the relative impedance-weighted demand from all origins.

This consideration has important implications. Consider the highest decay scenario $f_{1}(c_{ij})$. Under this scenario, Zone 1 – despite hosting a medium amount of physicians – captures the fewest physicians (133.469), compared to 166.781 at Zone 2, and 189.75 at Zone 3. Why? Zone 1 has the smallest population and is adjacent to Zone 3, the urban core. Its low impedance-weighted demand means $\kappa_{ij}^{S}$ allocates it fewer opportunities. By contrast, in the total-constrained case, Zone 1 fares better, capturing 35% of all physicians (compared to 27%).

As travel decay increases (e.g., from $f_{3}(c_{ij})$ to $f_{1}(c_{ij})$), competition becomes less diffuse. Zone 2, with the largest population, claims the most opportunities under $f_{3}(c_{ij})$. But under lower decay, Zones 1 and 3 draw relatively more opportunities from Zone 2. For instance, Zone 1 gains 6% more from Zone 2 in $f_{1}(c_{ij})$ than in $f_{3}(c_{ij})$. This shift reflects a drop in $\kappa_{2,2}^{S}$ of 14%, reflecting Zone 2’s decreasing hold on its own opportunities as other zones gain accessibility ‘parity’.

This dynamic reveals how $\kappa_{ij}^{S}$ embeds both travel impedance and population competition. Unlike the total constraint lowest decay scenarios that allocate evenly (as localized supply and demand are assumed to be unknown), the singly-constrained case reflects competition’s influence on allocation.

In this way, the consideration of constrained accessibility *per capita* may be clarifying. Often, accessibility values are reported as raw scores without considering the population potentially accessing them. But, as we introduced constraints, these constrained accessibility values can be normalized using anything that is relevant to the zone. In Table 8, we present per capita accessibility for the numeric example simply in units reflecting the number of physicians accessible per population at each zone. Notably, these per capita rates are equivalent to the 2SFCA values.

**Table 8:** Simple system: Singly-constrained accessible opportunities per capita.

Origin	Population (10k)	v${\;\;}_{\text{i}}^{\text{S}}$		
		f_1_ (c${\;\;}_{\text{ij}}$) = 1/c${\;\;}_{\text{ij}}^{3}$	f_2_ (c${\;\;}_{\text{ij}}$) = 1/c${\;\;}_{\text{ij}}^{2}$	f_3_ (c${\;\;}_{\text{ij}}$) = 1/c${\;\;}_{\text{ij}}^{0.1}$
		units: *physicians per capita*	units: *physicians per capita*	units: *physicians per capita*
1	4	33.367	30.564	24.712
2	10	16.678	18.510	24.188
3	6	31.625	30.442	24.879

This simple example was constructed so that the regional average equals 24.5 physicians per 10,000 people. As distance decay decreases and becomes *relatively* uniform (all zones can reach all zones), the effect of population drives the proportional allocation of opportunities. Consequently, per capita accessibility values begin to stabilize to the regional per capita average (e.g., in the highest distance decay $f_{1}(c_{ij})$, per capita values are all ~24 physicians accessible).

This convergence mirrors the trend in the total-constrained opportunity case, where accessibility values approach a third of the 490 physicians under the lowest-decay unfettered mobility scenario $f_{3}(c_{ij})$. In both cases, the balancing factors (*K*^*S*^ and *B*_*j*_) act as averaging mechanisms but at different scales. As distance decay becomes *relatively* more uniform, the role of remaining variables (i.e., total population or opportunities) drive the proportional allocation differences. In the total-constrained case, this is the proportion of opportunities relative to the regional opportunities, and in the case of the single opportunity constrained case, this is the population at a zone relative to the regional population.

#### 7.4.2 Singly-constrained accessible population: Market availability.

Similar to Eq 27 in transposing the origins and destinations, we can define a *singly-constrained* measure of market potential that preserves the known population (i.e., the mass weight at the origin $W_{i}^{\left(1\right)}$ is now represented by *O*_*i*_). In its per-capita expression, i.e., equivalent to 2SFCA, this constrained concept of market potential has been used to express “facility crowdedness” as in Wang [135].

The underlying spatial interaction model is now the production-constrained model version of Eq 8, and our market potential measure $M_{j}^{S}$ becomes:

$$M_{j}^{S}=\sum_{i}A_{i}O_{i}W_{j}^{\left(2\right)}f(c_{ij})$$ (35)

In this variant, the measure is singly-constrained by the population *by origin* (i.e., *O*_*i*_), like Eq 11 from Wilson’s framework:

$$\sum_{j}M_{ji}^{S}=O_{i}$$ (36)

And the corresponding balancing factor, as per Wilson, is:

$$A_{i}=\frac{1}{\sum_{j}W_{j}^{\left(2\right)}f(c_{ij})}$$ (37)

Following the same logic as in the preceding section on total-constrained market potential, one arrives at the following expression of accessible population $M_{j}^{S}$ being the product of proportionally allocated (${\hat{\kappa }}_{ji}^{S}$) population:

$$M_{j}^{S}=\sum_{i}{\hat{\kappa }}_{ji}^{S}O_{i}$$ (38)

with:

$${\hat{\kappa }}_{ji}^{S}=\frac{W_{j}^{\left(2\right)}f(c_{ij})}{\sum_{i}W_{j}^{\left(2\right)}f(c_{ij})}$$ (39)

As well, the single (population) constraint in Eq 36 ensures that the total constraint (e.g., $\sum_{j}M_{j}^{S}=\sum_{i}\sum_{j}M_{ji}^{S}=O$) is maintained.

With these constraints, $\frac{M_{j}^{S}}{O}$ can be interpreted as the proportion of the total population serviced by location *j*.

For the sake of brevity, we’ll move on to the doubly-constrained case.

### 7.5 Doubly-constrained accessibility

This accessibility case adopts the structure of the doubly-constrained spatial interaction model, where $V_{ij}^{D}$ flows are constrained by both origin populations *O*_*i*_ and destination opportunities *D*_*j*_. That is, the resulting accessibility outflow from each origin must match the origin’s population demand, and the resulting accessibility inflow to each destination must match the number of opportunities supplied:

$$\sum_{j}V_{ij}^{D}=O_{i}\text{ and }\sum_{i}V_{ij}^{D}=D_{j}$$ (40)

Because results are made to match values in both marginals, the results cannot be interpreted as a traditional summary at *i* or *j* (e.g., “opportunities accessible from *i*”), as evidently those sums simply reproduce themselves. Instead, the meaningful unit of analysis is the *ij* flow itself.

This distinguishes doubly-constrained accessibility from the total and singly-constrained cases discussed previously. In those cases, only one side of the interaction – either the total marginal or opportunity/population marginal – was constrained, while the other was treated as a demand/supply weight (e.g., *D* or *O* for total-constrained and $W_{j}^{\left(2\right)}$ or $W_{i}^{\left(1\right)}$ for singly-constrained).

By contrast, the doubly-constrained model assumes both the demand (population) and the supply (opportunity) are known and bounded, and allocates flows accordingly. This makes it less suitable for traditional accessibility analysis, namely because origin and destination masses often differ in kind and units. For instance, the number of people accessing an opportunity, such as a park, may be known, but the capacity of each park is not. A doubly-constrained approach may make conceptual sense if the “potential” should be contained only in the flows themselves, meaning the units of population and opportunities are comparable, have a one-to-one correspondence or are otherwise paired (e.g., job per worker, student per school seat, or vaccine doses per person). Mathematically, this model requires the simultaneous imposition of both the population- and opportunity- constraints in the preceding singly-constrained variants (Eq 28 and Eq 36), namely the sum of population in all origins should match the sum of opportunities in all destinations (Eq 41):

$$\sum_{i}O_{i}=\sum_{j}D_{j}$$ (41)

As before, the simultaneous imposition of both constraints ensures the total system constraint is maintained i.e., $\sum_{i}V_{i}^{D}=\sum_{i}\sum_{j}V_{ij}^{D}=D$ remains equal to the total number of opportunities in the region *O*.

The doubly-constrained accessibility measure $V_{ij}^{D}$ takes the form of the production-attraction (doubly-constrained) spatial interaction model as follows:

$$V_{ij}^{D}=A_{i}B_{j}O_{i}D_{j}f(c_{ij})$$ (42)

where the corresponding balancing factors *A*_*i*_ and *B*_*j*_, as per Wilson, are:


$$\begin{array}{c} A_{i}=\frac{1}{\sum_{j}B_{j}D_{j}f(c_{ij})}\\ B_{j}=\frac{1}{\sum_{i}A_{i}O_{i}f(c_{ij})} \end{array}$$

Calibration of the two sets of proportionality constants is accomplished by means of iterative proportional fitting, whereby the values of *A*_*i*_ are initialized as 1 for all i to obtain an initial estimate of *B*_*j*_. The values of *B*_*j*_ are used to update the underlying $V_{ij}^{D}$ matrix, before calibrating *A*_*i*_. This process continues to update *A*_*i*_ and *B*_*j*_ until a convergence criterion is met [66, see p. 193-195].

The doubly-constrained model completely distributes origin populations to destination opportunities according to travel impedance and supply-demand balance. This ensures that: summing $V_{ij}^{D}$ across *j* returns *O*_*i*_; summing across *i* returns *D*_*j*_. Thus, aggregating over *i* or *j* yields only the known constraints. In this way, a per-capita form (e.g., $V_{i}^{D}/O_{i}$) is not meaningful–since the output already reflects population-normalized allocation. As such, the *ij* matrix $V_{ij}^{D}$ is the only interpretable output.

We could define the proportional allocation factor $\kappa_{ij}^{D}$ such that:


$$\kappa_{ij}^{D}=\sum_{j}\frac{1}{\sum_{j}B_{j}D_{j}f(c_{ij})}\frac{1}{\sum_{i}A_{i}O_{i}f(c_{ij})}O_{i}f(c_{ij})$$

and represent $V_{ij}^{D}$ as equal to $\kappa_{ij}^{D}D_{j}$, allowing the analyst to understand the proportional allocation of *D*_*j*_s to each *ij* flow.

Following this logic, the market potential from $M_{ji}^{D}$ is effectively equivalent to $V_{ij}^{D}$, but can be read with a different interpretation: the opportunities accessed from *j* at an *i* vs. the population accessed from *i* at a *j*. The inputs of ‘opportunities accessed’ and ‘accessed population’ can already be interpreted as inherently being sensitive to both opportunities and population.

To calculate doubly-constrained accessibility using the toy example, the interpretation of the population data and the counts of the opportunity data must be reworked. Namely, a count of physician *capacity* per destination *D*_*j*_ is needed instead of simply the number of physicians. We also need to be able to clearly state that the population is the *capacity* of the origin to interact with opportunities *O*_*i*_, i.e., the count of people seeking opportunities.

This adjustment to the example is summarised in Table 9. With the population (in units of 10,000s of people seeking physicians) and the opportunities (in units of 10,000s of physician-capacity) per zone. For the population, we leave this unchanged numerically, but we now must keep in mind that each person interacts with one unit of physician capacity. The number of providers per destination is, however, revised to represent physician capacity, scaled approximately from the original number of physicians used in previous cases (Table 2). The system-wide PPR is now 1 (recall: the unmodified example’s system PPR is 24.5).

**Table 9:** Modified simple system with three zones reflecting matched population and opportunities. Population is in 10,000 persons and opportunities in 10,000 of physician-capacity.

ID (i or j)	Population	Opportunities
1	4	7
2	10	5
3	6	8

We keep the same zonal cost matrix and travel impedance functions for three types of travel behaviour as before (Table 3 and Eq 18).

And with these modifications to the example, our objective is slightly different: to predict the flows from *j* knowing that the amount of physician-capacity at each *j* must be preserved and all flows to *i* should match the number of people at *i*, under different travel behaviour scenarios. Put another way, we’re interested in the *ij* flows assuming we already know accessibility at each *i*. The highest decay travel behaviour scenario ($f_{1}(c_{i}j)$) is presented in Table 10.

**Table 10:** Doubly-constrained accessible opportunities assuming highest travel decay in the modified simple system.

	Origin ID	Destination ID			sum
		1	2	3	
	1	3.235859	0.01032226	0.7556568	4
	2	2.132602	4.95932483	2.9044391	10
	3	1.631539	0.03035291	4.3399040	6
Sum	—	7	5	8	—

As shown in Table 10 for the highest-decay scenario $f_{1}(c_{ij})$, accessibility is no longer meaningfully represented as zonal summaries like $V_{i}^{D}$ or $M_{j}^{D}$, since these values reproduce the original constraints, i.e., $V_{i}^{D}=O_{i}$, hence the physician-capacity accessible for Zones 1, 2, and 3 are 4.002, 9.996, and 6.002. The usefulness of the doubly-constrained measure lies in the interpretation as $V_{ij}^{D}$ values; the number of opportunities from zone *j* allocated to populations in zone *i* is shaped by both mass and travel impedance.

To illustrate this concept, consider the results for Zone 2 (recall: a suburban type zone, with a higher population, lower amount of opportunities, and relatively remote). As shown in Table 11, its intrazonal flow (i.e., $V_{22}^{D}$) declines as travel impedance decay decreases – from 4.959 under $f_{1}(c_{ij})$ to 2.667 under $f_{3}(c_{ij})$, out of the ~10 opportunities allocated to Zone 2 (a population of 10).

**Table 11:** Doubly-constrained accessible opportunities at Zone 2 for all travel decay groups in the modified simple system.

Dest.	Population at 2 (units: *people in 10,000s*)	Opportunities (units: *capacity in 10,000s*)	V${\;\;}_{\text{ij}}^{\text{D}}$		
			f_1_ (c${\;\;}_{\text{ij}}$) = 1/c${\;\;}_{\text{ij}}^{3}$	f_2_ (c${\;\;}_{\text{ij}}$) = 1/c${\;\;}_{\text{ij}}^{2}$	f_3_ (c${\;\;}_{\text{ij}}$) = 1/c${\;\;}_{\text{ij}}^{0.1}$
			units: *physician-capacity in 10,000s*	units: *physician-capacity in 10,000s*	units: *physician-capacity in 10,000s*
1	10.000	7.000	2.133	2.272	3.411
2	10.000	5.000	4.959	4.766	2.667
3	10.000	8.000	2.904	2.958	3.919

Following the intuition discussed in the singly-constrained opportunity case, as decay decreases, the mass effects (effect of the population and opportunities magnitudes) become relatively more dominant in the spatial allocation.

Accessibility is conventionally presented as a zonal value, not a flow. However, in the doubly-constrained case, since we force the allocation of zonal population demand and zonal opportunities supplied to be paired and allocation to be proportional, $V_{i}^{D}$ is simply the number of opportunities that matche our known population at *i*. Following the logic of the family of accessibility measures, in the doubly-constrained case, $V_{ij}^{D}$ flows are the only relevant unit of analysis: spatial proportional allocations between population and opportunity capacity. Furthermore, $V_{ij}^{D}$ and its transposed counterpart $M_{ji}^{D}$ are structurally identical, differing only in interpretation (referring to $\kappa_{ij}^{D}$ and ${\hat{\kappa }}_{ij}^{D}$): one reflects the proportional allocation of opportunity to population flows; the other, population to opportunity flows.

$V_{ij}^{D}$ are also mathematically equivalent to Wilson’s spatial interaction flows. And, as Wilson [43] explicitly noted, origin and destination weights defined in the spatial interaction model *can* be defined using any unit. However, the focus of these models has typically been on *ij* flows, often calibrated using trips (i.e., outbound and inbound trips, inherently in the same units). With the family of accessibility measures, it is made clear that we are working in units of opportunities and population.

And as mentioned, these units are often misaligned. For instance, we may not know how much park space, grocery area, or childcare capacity is accessible per person. When they do align, such as people to physician capacity, we would be modeling realized access flows based on known quantities of *those that interact* and the *interacted*. In such cases, the traditional accessibility question can be seen to be already answered by the known information (i.e., how many opportunities can be reached by a zone? Answer: the number of people at that zone). For this reason, we do not foresee the doubly-constrained measure being widely used in accessibility analysis, as the literature has largely focused on questions of ‘potential’, not on predicting flows of realized access.

## 8 Conclusions

In this paper, we examined the historical and mathematical commonalities between spatial interaction models and place-based accessibility measures. As accessibility research evolved largely influenced by Hansen [10], researchers in the field neglected the proportionality constant that was originally present in gravity-based models, and is still present in spatial interaction modeling. This work has demonstrated theoretically, and through a simple numeric example, that by reintroducing Wilson’s system constraints and defining associated balancing factors and proportional allocation factors, we can derive a unifying family of accessibility measures that reintroduces tangible units to the resulting values. These values may be more intuitive for the purpose of analysis and comparison.

To summarize the contributions of this work, first, we place the popular Hansen-type accessibility measure [10] within this family of measures as an “unconstrained” case, demonstrating that resulting values cannot be directly compared across different travel scenarios without ad-hoc adjustments. We then show how applying a total constraint balances the units and produces a statistically averaged solution that converges to the regional average for each zone as the decay effect decreases. In other words, the total-constraint model could be a more interpretable alternative for the unconstrained case if population-competition is not relevant and one is interested in capturing the maximum *potential*; specifically, if there is a fixed number of opportunities in the region, and if it makes sense to assume that people accessing proximate opportunities leave fewer for others, *without* considering the population size at the origins.

We then introduced the singly-constrained case, which *does* take into account the population size at the origin in the allocation of opportunities. It is also mathematically equivalent to the spatial availability introduced in Soukhov et al. 2023 [129]. In this case, all accessibility values are fixed to sum to a known zonal opportunity-size value (implicitly, the regional total of opportunities), but they are not required to sum to any population-based values at the zone or regional level. The singly-constrained model could be useful if regional competition is a factor and if the acknowledgment that only a finite number of opportunities can be allocated from each destination (with those allocations distributed based on origin population size) is suitable. We also introduce an ‘accessible’ PPR (e.g., opportunities per capita), calculated by dividing each accessibility value by the zonal population. To clarify, this per capita expression of the singly-constrained case is equivalent to the 2SFCA [17,44], hence linking this literature back to spatial interaction principles.

Lastly, the doubly-constrained case is introduced. In this case, the sums must equal both the regional total and ensure that no zone allocates more opportunities than it has available. Specifically, accessibility values for each i−j pair must be a proportion of the zonal opportunity and population values simultaneously. For example, the accessibility at Zone 1 must equal the sum of opportunities from Zones 1, 2, and 3, as well as the sum of the population at Zone 1. Satisfying the double constraint means the opportunities and population data must match one-to-one, so working with the accessibility i−j pair value should be of research interest.

Building on Wilson’s [43] foundational work, this paper proposes a unified framework for analyzing gravity-based accessibility. By reintroducing Wilson’s proportionality constants, the family of constrained accessibility measures restores measurement units to accessibility estimates. This enhancement provides a more interpretable, consistent, and theoretically grounded basis for accessibility analysis, which could help advance the adoption of accessibility-oriented planning.

While this work exclusively focused on top-down gravity-based accessibility measures, there have been recent developments in the accessibility literature that include person-based approaches that are time-sensitive [22,136], behavioural [137,138] or utility-based [139,140]. While gravity-based measures of accessibility still dominate the applied literature, future work could further explore how these ideas of proportionality constants and balanced units could also help inform these and other new modeling approaches.
