# Supplementary material for: A family of accessibility measures derived from spatial interaction principles
Source: PLoS One. 2025 Nov 14;20(11):e0335951. doi: 10.1371/journal.pone.0335951 (PMC12617948; doi:10.1371/journal.pone.0335951)
Supplement: Attachment — Submitted filename: reviewer_respose_letter_V1.pdf [file pone.0335951.s001.pdf]

Response to reviewers

Dear editors,

We appreciate the valuable comments from the reviewers. We have done our best to address all comments and suggestions as much as possible, which we believe has significantly improved the paper. We address each comment of the reviewers point by point (as follows), but we would also like to note three significant points in response to the reviewers' recommendations.

- We have substantially shortened the paper from 34 to 28 pages. This involved streamlining the manuscript's text, removing equations and text redundancies where possible, especially in the "Family of Accessibility" section.
- We have also edited the text to more clearly articulate the paper's main contribution and to make each section's purpose clearer.
- While we agree with Reviewer #2's comment that suggested including an empirical example to demonstrate the real-world applicability of our proposed measures, we were not able to include such a case study in the paper due to manuscript length limits. As such, we've toned down language concerning real-world applicability and instead point to how the theoretical clarity of the resulting values can be helpful practically. An empirical case study will be left for future work.

As a whole, we believe the paper is more consistent after heeding the reviewer's comments. We hope you and the reviewers will agree. Please see below the point-by-point replies, with our response in black font and the reviewers comments in blue.

Response to Reviewer 1

Thanks for the opportunity to review a paper that handles an interesting topic. I think the major contribution of the paper lies in 1) combining the concept of accessibility and spatial interaction modeling and 2) classifying the numerous accessibility measures into four categories based on the constraints. While the topic is really interesting and handles the important topic, I would like to ask the authors to revise the paper due to the following reasons.

Most importantly, the manuscript is too lengthy and verbose. I am having a hard time evaluating the manuscript since the length is about 30 pages. Given that the typical length of the paper is about 10 pages, I would like to request a summary of the key points for better deliverability.

Thank you for this point. We've significantly revised the manuscript to make it shorter and to ensure we articulate the key points of each section:

- We've substantially reduced the length of the manuscript from 34 to 28 pages (without references).
- We've removed tangential details and minimized the equations where possible, particularly in the "Early Research" section.
- We've streamlined the manuscript's text–to make each sections purpose more clear. The introductions and conclusions of sections now have a better flow.
- We've removed redundancies where possible, especially in the "Family of Accessibility" section.
- We've more clearly highlighted the paper's main contribution: namely, a review of the shared origins of spatial interaction modeling and accessibility, how their interpretations have diverged over time, and the introduction of a family of accessibility measures grounded in spatial interaction principles.

The key contributions of the manuscript are highlighted in the introduction of the paper (see paragraph starting at line 48) and can be summarised as follows: - First, it explicitly links accessibility to spatial interaction through their shared intellectual foundations (pre- Hansen (1959)), highlighting how contemporary accessibility research has diverged and showing the mathematical, intuitive, and interpretative advantages of preserving units through constrained formulations, as done in spatial interaction modeling. - Second, it introduces 'constraints' to accessibility measures grounded in spatial interaction principles.

Related to the first issue, I am not sure about the point that the authors explain the details of Newtonian's roots and early research on human spatial interactions from Ravenstein to Stewart. Of course, I think mentioning those contents is beneficial, but it is hard to get the key point given that it is verbose.

The main aim of these two sections is to demonstrate how Newton's gravitational equation (with its proportionality factor G) inspired early research on human spatial interactions. The majority of early researchers relied on the underlying proportional mass/distance relationship to model human spatial interaction, but did not use G–except for Stewart (1947). However, Stewart didn't have an answer to what G should be, just that it is needed to move from a relationship of proportionality to one of equality.

Stewart is especially important because Hansen (1959) later adopted it into his equation but omitted both the G (i.e., kept it set to 1) and was quiet on the broader issue of moving from proportionality to equality.

We've revised these sections. We hope we've sufficiently clarified these points and the purpose of these sections in the process.

Third, I believe the main contribution of this paper lies in classifying the accessibility measures into four groups. However, I also had a hard time finding the definition of those four groups. Given that the research articles aim to reveal previously unknown facts, the literacy level of this manuscript is relatively low. Additionally, the introduction of too many equations makes it difficult to establish connections between them. Therefore, I recommend that the authors revise and reconstruct the manuscript.

Fair points, thank you for raising them. To assist with comprehension, we now clearly summarize all four groups of constrained measures at the start of the "A Family of Accessibility Measures: From Proportionality to Equality" section. These definitions build on the preceding sections, which trace how spatial interaction modeling followed the Newtonian analogy while neglecting G, how Wilson shifted to an entropy-maximisation formulation without requiring an empirical G (but instead, a constraint that functions to tether the results to system knowns), and how the place-based accessibility literature has largely overlooked this solution. The main section then presents the mathematical formulations and a toy example to illustrate the full family of measures.

Furthermore, regarding the equations – 10 equations have now been removed because they did not directly further the argument of the section or are redundant. For instance, some equations in "Early research on human spatial interaction" have been removed, and rewritings of Wilson's general formula from "Wilson's family of spatial interaction models" section have been removed.

Response to Reviewer 2

This manuscript presents a theoretically motivated and conceptually rich framework that bridges spatial interaction modeling and accessibility measures. The authors introduce a family of accessibility metrics grounded in Wilson's spatial interaction models and argue convincingly for the reintroduction of interpretability and measurement units in accessibility analysis. The historical tracing of accessibility theory development and the synthesis of divergent literature streams are timely and valuable contributions to the field.

1. While the authors provide a detailed numerical example, the manuscript lacks any empirical case study or application to real-world accessibility data. This limits the reader's ability to evaluate the practical utility and robustness of the proposed framework. The authors can draw inspiration from several studies such as "Factoring in temporal variations of public transit-based healthcare accessibility and equity" to better understand this concept.

Thank you for this point. We agree, the inclusion of an empirical example would demonstrate the real-world applicability of the measures. However, the length of the current document is an issue, so the inclusion of additional pages isn't feasible–especially given the concern over

paper length that was raised by Reviewer #1 and how we've reduced the manuscript by six pages in response to these concerns.

As well, we put great thought into how the manuscript could be restructured to included an additional empirical example. However, many of these ideas are not possible given the manuscript's current format. For instance, even if we replaced the toy example with an empirical example, it would still add quite significant additional length (i.e., explanation of data, additional space needed for tests). Further, a single empirical example is not sufficient to cover all cases and variants of the family.

As such, we've worked to clarify that the contribution of this work which is primarily theoretical and methodological. We've also tempered earlier claims about the measure's real-world applicability, instead allowing readers to consider potential uses and the potential value of reuniting accessibility measures with interpretable units. While we believe these measures usefulness would be highlighted through real-world examples–we plan to explore these in future publications which make this contribution more practically clear (i.e., where we may have enough space to develop and explore an empirical example without shortchanging the historical context).

In fact, this comment inspired action. We are currently in conversation with the editor about the possibility to submit a separate paper demonstrating the proposed measures with an empirical case study. We really appreciate the perspective this comment added.

2. The manuscript frequently shifts between concepts such as "potential for spatial interaction," "accessibility," and "access," especially in the discussion of the doubly constrained model. However, the terminological distinction remains conceptually blurry.

Completely agree, the use of these words–especially in the doubly constrained model subsection should be tuned up. In response, we've substantially revised this subsection. Namely, we've removed the discussion about the meaning of "potential", and instead focus on the embedded assumption of the doubly constrained measure i.e., how it simultaneously considers both origin-side and destination-side constraints–or that all people are matched with opportunities, and vice versa. We discuss how this is not a frequent situation in place-based accessibility analysis (i.e., we don't know how much parkland space capacity is matched to each person).

3. The historical literature review is valuable but disproportionately long. Pages are devoted to recapping classical gravitational thinking, while the more innovative contributions of the paper (e.g., balancing factor reinterpretation) are condensed. The authors should also focus on more recent publications such as "Evaluating temporal variations in access to multi-tier hospitals using personal vehicles and public transit: Implications for healthcare equity" instead of older ones.

Thank you for these valuable points. In response, we've made significant efforts to streamline the introductory and review sections (i.e., before *"A family of accessibility measures: from proportionality to equality"* section), to ensure a better balance with the paper's original contributions. These sections are now 12 pages instead of the 15 pages in the previous version,

and now include additional text at the end and start of each section to better summarise and bridge the ideas. We also removed 6 equations that we viewed as non-essential in these earlier sections along with removing redundant/tangential text to reduce the overall length while still covering all necessary literature and the over 70 papers that cite both Wilson (1971) and Hansen (1959).

We agree that these early sections must not overshadow the other core contributions. Accordingly, we also improved the clarity of the *"A family of accessibility measures: from proportionality to equality"* section by streamling the *"Toy example setup"* subsection and significantly improving the flow of the discussion of toy example results for each case. We reduced redundant text and removed an additional 4 equations. We believe the paper now strikes a more appropriate balance between historical context and novel insights.

Regarding the suggested inclusion of more recent work, we have now added "Evaluating temporal variations in access to multi-tier hospitals..." to the Introduction as an example of recent work. Furthermore, while it offers important insights on temporal accessibility and equity, the review section of our work is scoped specifically to place-based accessibility measures that engage with spatial interaction models. As such, we found this work outside the direct narrative arch of the work's earlier section. Nonetheless, we've also included a brief acknowledgement of the broader literature on temporal considerations in accessibility approarches in the concluding paragraph of the Conclusion section (see response to the following question).

We hope these revisions address your concerns and improve the clarity and balance of the manuscript. Thank you again.

4. The paper pays limited attention to recent methodological developments such as time-sensitive, network-based, or multi-modal accessibility models, as well as behavioral or utility-based approaches.

Thank you for this fair point. In the revised manuscript, we state explicitly that our focus is on place-based accessibility, which remains widely used in practice and prominent in the literature, as well as Wilson's spatial interaction model which is also popular in spatial interaction modeling to this day. Given the broad scope of the paper and space constraints, incorporating additional detailed discussions of novel approaches is challenging, and somewhat tangential.

However, we do acknowledge some utility-based approaches within the spatial interaction literature. For example, on page 11 "The third subset of the spatial-interaction focused literature, depart from Hansen's definition, aligning instead with microeconomic or utility-based interpretations of potential spatial interaction e.g., Morris et al. (1979) and Leonardi et al. (1984)".

Our point with the historical review section is to demonstrate how even the papers that *do* cite both Wilson and Hansen do not operationalise Wilson's balancing factors. Utility-based approaches take a different direction–not based explicitly on Newtonian gravitation–but also not based on Wilson's balancing factors. Indeed recent developments such as time-sensitive, network-based, or multi-modal accessibility models, as well as behavioral or utility-

based approaches take on new approaches, but we believe these are tangential to our scope but are valuable to mention.

In response, we've added a paragraph at the end of the conclusion that mentions this explicitly: "That said, there have been more recent developments in the accessibility literature which include novel approaches such as person-based approaches, including those that are time-sensitive (Yang et al. 2024; Braga 2024 et al. 2023), behavioural (Kar et al. 2024; Lu et al. 2014), or utility-based (Guzman et al. 2023; Ben-Akiva and Lerman 1985), that are not based on top-down approaches such as Wilson's model formalized through the entropy-maximisation procedure. Future work could further explore how these ideas of proportionality constants and balanced units could help inform new modeling approaches."

5. The authors argue that the constrained models restore interpretability by attaching meaningful units (e.g., number of people or opportunities). However, this is not substantiated with user- or practitioner-oriented tests.

Thank you for giving us the opportunity to clarify this point. The argument that constrained models attaches meaningful units to accessibility measure is theoretically demonstrated in the paper, and can be checked with careful examination of the equations. Nonetheless, we also believe this is demonstrated with the explanation of the toy example used in the paper.

First, this idea is brought up more concretely on page 5, when discussing Stewart (1948)'s use of his proportionality constant G: "In other words, the addition of G shifts results from being abstract indicators of potential (i.e., $\frac{\text{people}^2}{\text{distance}^2}$) to having interpretable units grounded in consistent, albeit still abstract, quantities (i.e., units of demographic force)."

Later, we develop this point deeper when discussing Hansen-type accessibility, and the impact of not having a proportionality constant: pg. 6: "Furthermore, working with a proportional relationship generates fundamental issues in comparability between and, arguably within, studies. Namely, accessibility estimates have no fixed unit, rendering them sensitive to the choice of impedance functions. For instance, if travel cost d_{ij} is measured in meters, then when the travel impedance function $f(d_{ij})$ equals $d_{ij}^{-\beta}$, the resulting S_i has units of opportunities per metres$^{\beta}$. However, when $f(d_{ij})$ is set to equal $e^{-\beta d_{ij}}$, the units become opportunities per $e^{\beta \text{metres}}$. Such variation impairs comparability across analysis and obscures the meaning of accessibility scores, making them difficult to understand and communicate without post-hoc treatment."

And to solidify this point, again more theoretically but still practically, we develop and solve the toy example. We discuss the advantage of interpreting the units when constraints are used, and the challenge of doing so across toy example scenarios in the case of the unconstrained example.

Namely, concerning the unconstrained example on pg. 17 we write: "For example, Table 4 shows V_i^0 under each decay function. Comparing across decay types is meaningless in absolute terms. For instance, the difference in zone 1 (edge of urban core)'s accessibility under f_3 vs f_1

is 370.92, but in what units? These two values are a product of different impedance functions (*physicians-minute*$^{-3}$ and *physicians-minute*$^{-0.1}$), making the comparison uninterpretable (and arguably incorrect). The fundamental uninterpretability of what is a *opportunity-weighted-travel-impedance* unit remains."

Building on this idea, we discuss the interpretive clarity in the total constrained numeric example on pg. 19: "Compared to the unconstrained case, values now sum to the known regional total D, allowing interpretation of absolute and relative differences across zones and travel scenarios. For example, in the highest decay case, Zone 1 (Urban Edge) captures an intermediate number of physicians (172.06), like in the unconstrained accessibility case. However, unlike in the unconstrained case, we can say that this value is out of the 490 physicians in the region, which allows us also to deduce that zone 1 captures 1.31 and 0.92 times more than zone 2 and 3."

We also go on to write on pg. 19: "One can also directly compare values at a specific zone, across travel impedance scenarios, due to the consistent units. As the decay scenario decreases, all zones become more accessible to each other and the differences between pairs diminishes (i.e., in $f_3(c_{ij})$ each zone captures close to an average amount of physicians, a third of 490 or ~163). In terms of proportional magnitude, this can also be observed in the unconstrained measure for this scenario. However, for the total constrained measure, this plateauing of results have meaning. In fact, each zone is allocated an average of the total amount in the region, as a result of the total constrained proportional allocation factor."

Through the discussion of the toy example, we hope we make clear the interpretative clarity that using the constrained framework offers.

6. Some sections suffer from repetition and overly technical language without summarizing takeaways (e.g., Table 1 is helpful, but the text before and after it repeats much of the same information).

A fair point. We've streamlined redundancies throughout the manuscript, especially in the "A family of accessibility measures" section. Specifically regarding table 1, we've gone back-and-forth on it and ultimately opt to include it as a helpful and more plain-language summary. We also provide more condensed descriptions in text, namely:

"The proportional allocation constant κ takes the form of a balancing factor that varies depending on the constraints applied. Each member of the accessibility measure family is defined by the constraints used, and can be grouped into the following four cases:

1. **Unconstrained Case (V_i^0, M_j^0)**

- Equivalent to Hansen's (Hansen 1959) and Reilly's (Reilly 1929) original formulations; the status quo of accessibility modelling.
- No balancing factors applied; units are in "opportunities-by-impedance" for V_i^0 or "population-by-impedance" for M_j^0.

- No constraints are applied, so values reflect proportionality only and are not calibrated to known system totals.

2. **Total Constrained Case (V_i^T, M_j^T)**

- Applies a total proportional allocation factor (κ_{ij}^T, $\hat{\kappa}_{ji}^T$) based only on the total marginal (green box in Figure 2) i.e., total number of opportunities or population in the system. This ensures the sum of all values in the system match the total marginal.
- Units of V_i^T: accessible opportunities from i, a value that is total constrained and linearly proportion to V_i^0.
- Units of M_j^T: accessible population from j, a value that is total constrained and linearly proportion to M_j^0.

3. **Singly Constrained Case (V_i^S, M_j^S)**

- Applies singly-constrained proportional allocation factors (κ_{ij}^S, $\hat{\kappa}_{ji}^S$) based on Wilson's balancing factors (B_j, A_i) to preserve either the destination-side or origin-side marginal totals (blue and red boxes in Figure 2) i.e., the number of opportunities or population at each zone. Reflects how the literature calculates competitive accessibility.
- Units of V_i^S: accessible opportunities from i, a value that is the sum of opportunity supply flows allocated proportionally based on demand at i. Mathematically equivalent in per-capita form to 2SFCA (Luo and Wang 2003).
- Units of M_j^S: accessible population from j, a value that is the sum of population demand flows allocated proportionally based on supply at j.

4. **Doubly Constrained Case (V_{ij}^D, M_{ji}^D)**

- Constrained on both origin and destination sides using both A_i and B_j simultaneously, which can also be expressed as proportional allocation factors (κ_{ij}^D, $\hat{\kappa}_{ji}^D$); equivalent in interpretation to Wilson's (Wilson 1971) doubly constrained spatial interaction model.
- Simultaneous application ensures both the destination-side *and* origin-side marginal totals are maintained (blue and red boxes in Figure 2).
- Interpretable only as ij and ji flows, since aggregation at i and j simply reproduces known totals. Represents 'interaction capacity' or 'realized access' serving as predictions of real interaction flows."

We hope this addresses your concern!

References

Hansen, Walter G. 1959. "How Accessibility Shapes Land Use." *Journal of the American Institute of Planners* 25 (2): 73–76. https://doi.org/10.1080/01944365908978307.

Luo, Wei, and Fahui Wang. 2003. “Measures of Spatial Accessibility to Health Care in a GIS Environment: Synthesis and a Case Study in the Chicago Region.” *Environment and Planning B: Planning and Design* 30 (6): 865–84. https://doi.org/10.1068/b29120.

Reilly, William J. 1929. “Methods for the Study of Retail Relationships.” 2944.

Wilson, A. G. 1971. “A Family of Spatial Interaction Models, and Associated Developments.” *Environment and Planning A: Economy and Space* 3 (1): 1–32. https://doi.org/10.1068/a030001.
