# Supplementary material for: A family of accessibility measures derived from spatial interaction principles
Source: PLoS One. 2025 Nov 14;20(11):e0335951. doi: 10.1371/journal.pone.0335951 (PMC12617948; doi:10.1371/journal.pone.0335951)
Supplement: Attachment — Submitted filename: reviewer_respose_letter_V2.pdf [file pone.0335951.s002.pdf]

Response to reviewers V2

Dear editors,

We appreciate the valuable comments from the reviewers on this second round of revisions. Changes to the manuscript are much lighter than the previous round, reflecting the quality of the manuscript as is and the lighter points raised by the reviewers.

We address each comment of the reviewers point-by-point, with our response in black font and the reviewers comments in blue.

Response to Reviewer 1

NA

Thank you for your efforts in the previous round of revision Reviewer 1!

Response to Reviewer 2

NA

Thank you for your efforts in the previous round of revision Reviewer 2!

Response to Reviewer 3

This is my first time seeing the paper, though I can see it has already been through a major revision. Looking at the previous comments and changes it looks like you have responded to the comments of the reviewers. The paper makes a valuable contribution in drawing together these different accessibility measures that many researchers know are somehow related but wouldn't be able to explain how. The review of literature included also adds value and will make this a valuable paper for people working in the field to read.

I was hoping not to have to ask for any further revision because I know it can be frustrating when a new reviewer is brought in after a revision has been made. However, reading your paper reminded me of Cesario (1977) who also discussed the interpretation of balancing factors from spatial interaction models as accessibility measures. I can't see that this is cited in the work, but given the nature of what you are doing and the historical approach taken in parts of your paper I think it should be included in the discussion.

Cesario, F. J. (1977). A new interpretation of the "normalizing" or "balancing" factors of gravity-type spatial models. Socio-Economic Planning Sciences, 11(3), 131-136.

Thank you for these positive comments and for the helpful suggestion regarding Cesario 1977. We have incorporated this citation in the section preceding the literature review Section 6 (i.e., "Accessibility and spatial interaction modelling: two divergent research streams"). Specifically, the final paragraphs of "Wilson's family of spatial interaction models" section 5 now read:

> *Wilson's work is notable for many reasons. Rather than relying on a universal constant like G or a scaling factor to balance units, Wilson's models calibrate interaction flows through known empirical constraints using principles of entropy maximisation. This results in interpretable, balanced (given the constraints), and unit-consistent ij flows. The balancing factors themselves have been subject to various interpretations–as terminal costs (Dieter 1962), weighted mean values (Kirby 1970), or as accessibility measures themselves ((Cesario 1977) as suggested in Wilson (Wilson 1971)) and as rents (Morphet and Shabrina 2023)–reflecting ongoing efforts to understand what these mathematical constructs represent behaviourally. In this way, the spatial interaction modeling tradition can be seen to have succeeded where accessibility modeling stalled. While Wilson's model produces results that are in units of flow tethered to the system of analysis, which facilitates such interpretations, Hansen-type measures (still widely used today in accessibility work) yield outputs that reflect proportional – not equal – relationships and typically lack interpretable units.*
>
> *Before demonstrating the derivation of family of accessibility measures that are sensitive to constraints (Section 7), in the next section we review how conceptually intertwined the spatial interaction modelling and accessibility literatures are, and where they began to diverge. This investigation sheds light on why accessibility research may have failed to adopt a comparable approach focused, until this paper.*

Response to Reviewer 4

The manuscript presents a theoretically rich and mathematically detailed framework that attempts to bridge spatial interaction modelling and accessibility analysis. While the topic is interesting and potentially valuable, I found the paper quite challenging to read due to its

length and the density of the mathematical exposition. The presentation is heavy and at times difficult to follow, which limits accessibility to a broader audience.

We appreciate the recognition that our work addresses an important theoretical gap. In the previous revision round, we substantially shortened the manuscript from 34 to 29 pages, streamlined the use of mathematical exposition, and clarified each section's contribution in response to concerns about length from other reviewers. This reviewer now reports to be satisfied with our changes in this round.

While we acknowledge you that the mathematical detail may be dense, we believe it is inherent to our contribution. Our manuscript establishes formal relationships between spatial interaction models and accessibility measures–specifically focusing on the use of the balancing factors. This manuscript offers a methodological and theoretical advancement that we view requires mathematical logic that matches what is common in the field of spatial interaction.

To address the general concern with reader navigation, we have enhanced the paper's overview (Section 1, final paragraphs). We now explicitly preview the paper's logical structure, explaining what each section accomplishes and how Sections 2-6 build the conceptual foundations for the derivations (core contribution of the paper) in Section 7. We believe this will help the readers navigate the text with clearer orientation. The following is the revised text:

> *The remainder of this paper proceeds as follows. Sections 2-4 trace the historical development of spatial interaction and accessibility research, beginning with Newtonian gravitational analogies and Carey (1858) (Section 2), moving through early researchers like Ravenstein (1885) to Stewart (1948) who theorised and formalized spatial interaction patterns (Section 3), and examining how the "gravity-based" accessibility approach in Hansen (1959) became the dominant approach in planning practice (Section 4). Section 5 presents the entropy-based family of spatial interaction models in Wilson (1971), explaining how the introduction of "constraints" (based on top-down known information as part of entropy-maximisation) produce interpretable, unit-consistent flow estimates. Section 6 explores why accessibility and spatial interaction literature diverged into separate branches despite their shared conceptual foundations. Section 7 constitutes the paper's core contribution: we derive a family of accessibility measures corresponding to different constraints applied–unconstrained, singly-constrained, and doubly-constrained cases–demonstrating how each yields zone-level accessibility values expressed in meaningful units (opportunities or population) rather than unit-inconsistent indices. We illustrate each measure with numerical examples that clarify the practical implications of different constraint assumptions. Section 8 concludes by discussing how these constrained measures can inform planning decisions and improve clarity in accessibility analysis.*
>
> *The key insight is that accessibility need not be unit-inconsistent. By building from spatial interaction theory's constraint framework rather than the gravitational analogy alone, we show how to construct accessibility measures that are simultaneously*

grounded in behavioural principles (the gravitational analogy) as well as being expressed in interpretable units (opportunities or population) that are sensitive to the known to the region through empirically-set constraints.

As well, we'd like to note that the manuscript already underwent extensive revision that included the addition of intuitive explanations before mathematical developments, removal/consolidation of tangential equations throughout to improve readability, and transitional text at the end of each section.

Together, we believe the addition to the Introduction and previous changes made should address the Reviewer's concerns. We believe the manuscript strikes a balance between readability and the detail offered by the mathematical expressions. We would welcome more specific and directed feedback from the reviewer regarding particular sections or derivations that remain unclear, as this would allow us to target improvements more effectively.

References

Carey, Henry Charles. 1858. *Principles of Social Science.* University of Michigan Library Digital Collections: In the digital collection Making of America Books. https://name.umdl.umich.edu/AFR1829.0001.001.

Cesario, Frank J. 1977. "A New Interpretation of the 'Normalizing' or 'Balancing' Factors of Gravity-Type Spatial Models." *Socio-Economic Planning Sciences* 11 (3): 131–36.

Dieter, Karl Heinz. 1962. "Distribution of Work Trips in Toronto." *Journal of the City Planning Division* 88 (1): 9–28.

Hansen, Walter G. 1959. "How Accessibility Shapes Land Use." *Journal of the American Institute of Planners* 25 (2): 73–76. https://doi.org/10.1080/01944365908978307.

Kirby, Howard R. 1970. "Normalizing Factors of the Gravity Model—an Interpretation." *Transportation Research* 4 (1): 37–50. https://doi.org/10.1016/0041-1647(70)90073-0.

Morphet, Robin, and Zahratu Shabrina. 2023. "Gravity Model Calibration by Rent." *UCL - Working Paper Series.*

Ravenstein, E. G. 1885. "The Laws of Migration Paper 1." *Journal of the Royal Statistical Society* 48 (2): 167–227.

Stewart, John Q. 1948. "Demographic Gravitation: Evidence and Applications." *Sociometry* 11 (1): 31–58. https://doi.org/10.2307/2785468.

Wilson, A. G. 1971. "A Family of Spatial Interaction Models, and Associated Developments." *Environment and Planning A: Economy and Space* 3 (1): 1–32. https://doi.org/10.1068/a030001.
